# Structural and dynamic origins of ESR lineshapes in spin-labeled GB1 domain: the insights from spin dynamics simulations based on long MD trajectories

**DOI:** 10.1038/s41598-019-56750-y

**Published:** 2020-01-22

**Authors:** Sergei A. Izmailov, Sevastyan O. Rabdano, Zikri Hasanbasri, Ivan S. Podkorytov, Sunil Saxena, Nikolai R. Skrynnikov

**Affiliations:** 1 Laboratory of Biomolecular NMR, St. Petersburg State University, St. Petersburg, 199034 Russia; 2 Department of Chemistry, University of Pittsburgh, Pittsburgh, PA 15260 USA; 3 Department of Chemistry, Purdue University, West Lafayette, IN 47907 USA

**Keywords:** Computational biophysics, Molecular biophysics, Proteins, Structure determination, Solution-state NMR

## Abstract

Site-directed spin labeling (SDSL) ESR is a valuable tool to probe protein systems that are not amenable to characterization by x-ray crystallography, NMR or EM. While general principles that govern the shape of SDSL ESR spectra are known, its precise relationship with protein structure and dynamics is still not fully understood. To address this problem, we designed seven variants of GB1 domain bearing R1 spin label and recorded the corresponding MD trajectories (combined length 180 μs). The MD data were subsequently used to calculate time evolution of the relevant spin density matrix and thus predict the ESR spectra. The simulated spectra proved to be in good agreement with the experiment. Further analysis confirmed that the spectral shape primarily reflects the degree of steric confinement of the R1 tag and, for the well-folded protein such as GB1, offers little information on local backbone dynamics. The rotameric preferences of R1 side chain are determined by the type of the secondary structure at the attachment site. The rotameric jumps involving dihedral angles χ_1_ and χ_2_ are sufficiently fast to directly influence the ESR lineshapes. However, the jumps involving multiple dihedral angles tend to occur in (anti)correlated manner, causing smaller-than-expected movements of the R1 proxyl ring. Of interest, ESR spectra of GB1 domain with solvent-exposed spin label can be accurately reproduced by means of Redfield theory. In particular, the asymmetric character of the spectra is attributable to Redfield-type cross-correlations. We envisage that the current MD-based, experimentally validated approach should lead to a more definitive, accurate picture of SDSL ESR experiments.

## Introduction

Over the last three decades, the field of structural biology has made a tremendous progress. This progress should be mainly credited to high-resolution X-ray crystallography and, to a lesser extent, solution-state NMR. More recently, cryo-EM microscopy became a major source of medium-resolution data. Furthermore, solid-state NMR emerged as a valuable addition to the repertoire of structure-solving techniques.

However, there are many important protein systems, which defy conventional structure-solving strategies. X-ray diffractometry is contingent on one’s ability to obtain a crystalline sample. NMR spectroscopy is limited by the size of the system. Generally, all techniques, including cryo-EM microscopy, tend to have difficulties with those systems that are highly inhomogeneous and/or highly dynamic. Many of the most important cellular systems fall in this category, e.g. chromatin, nuclear pore complex, cilia, etc. Some of the aberrant protein assemblies also have these characteristics, e.g. the so-called protofibrils, neurofibrillary tangles, etc. For those more challenging samples, valuable structural information can often be obtained by means of ESR spectroscopy.

Applications of ESR spectroscopy to protein samples usually rely on the popular spin-labeling reagent, (1-oxyl-2,2,5,5-tetramethylpyrrolinyl-3-methyl)-methanethiosulfonate (abbreviated MTSSL, shown below). The methanethiosulfonate group in MTSSL is selectively reactive toward thiols. Consequently, this compound achieves near-quantitative labeling of all solvent-accessible cysteine sites in a protein. Usually it is desirable to selectively label only one (or two) such sites. This is achieved by means of site-directed mutagenesis to introduce unique cysteines at the selected sites on the protein surface^1^. Following the conjugation with MTSSL, one obtains a modified cysteine residue (in what follows, this residue is called R1 in accordance with the established practice^2^). This modified residue carries the nitroxyl radical, which gives rise to a characteristic ESR signal in a form of triplet.

It has been recognized early on that the shape of the ESR spectrum depends on the dynamic status of nitroxyl probe. Generally speaking, if the probe experiences extensive motion (as seen in the laboratory frame of reference) and this motion is rapid then such probe produces sharper, more symmetric spectrum. Conversely, if local dynamics is restricted and the probe reorients slowly, then the spectrum tends to be broadened and asymmetric^3^. The observed ESR signal can, therefore, report on various local or global changes in the protein system insofar as these changes are reflected in the mobility of the nitroxyl probe.

The use of nitroxyl ESR lineshape as (phenomenological) reporter of changes in protein dynamics/structure led to many successful applications. For instance, this broad principle has been used to study conformational rearrangements in transmembrane receptors and transporters^4–6^, oligomerization of membrane proteins^7–9^, functional dynamics in enzymes^10–14^, molecular mechanisms of motor proteins^15,16^, etc. In doing so, many useful insight have been obtained by means of a qualitative comparison of ESR spectra obtained under different conditions. However, all along there has been a strong desire to move toward a more rigorous treatment. Early on, it has been acknowledged that R1 side chain contains five rotatable bonds and, therefore, much of the dynamics sensed by nitroxyl radical is caused by side-chain rotameric jumps. With this consideration in mind, Freed and co-workers implemented the SRLS (slowly relaxing local structure) model that seeks to separate internal motion from global dynamics^17,18^. This model provides an elegant parameterization of the problem, but does not necessarily reveal the exact origins of the motion, reflected in the spectra.

Hubbell and co-workers approached the problem by conducting extensive mutagenesis experiments^2,19,20^. They concluded that the shape of ESR spectrum is dependent primarily on the steric confinement of the R1 proxyl ring. As such, spin labels attached to the sites that are located in the loop regions are most mobile. Those that are attached to the solvent-exposed sides of α-helices or the outer edge of β-sheets are less mobile. Those that are semi-buried (confined by the elements of tertiary structure) are still less mobile. Finally, those that are fully buried (trapped in the protein hydrophobic core) are least mobile. Yet, mutagenesis experiments are not always clear-cut since any mutation may have certain unanticipated and unintended consequences. Occasionally the effect of mutations on ESR spectra does not match one’s intuitive expectations. Such discrepancies have been hypothetically attributed to the influence of backbone dynamics^2^.

Hubbell and co-workers also realized that internal mobility of R1 may depend not only on the mechanistic confinement factor, but also on certain more explicit interactions. They have suggested that “nonspecific hydrophobic packing” is one of the determinants of R1 dynamics. Indirect support for this hypothesis was provided by a series of ESR measurements in the presence of dioxane^21^. They have also hypothesized that mobility of R1 can be restricted by a non-standard hydrogen bond between sulfur atom and backbone amide HN^2,12,22^. Alternatively, they invoked weak hydrogen bond between sulfur atom S^δ^ and H^α^C^α^ group to explain the restricted character of side-chain motion, as well as similar unorthodox weak interactions formed by proxyl ring^20,23^. Presumed electrostatic interactions between side-chain carboxylic groups and the proxyl ring has also been discussed^20^. More generally, there has been an effort to identify specific interactions responsible for selection of rotameric states of R1^21,23,24^. In particular, it has been suggested that different rotameric states of R1 can help to explain the appearance of multicomponent ESR spectra (e.g. those spectra that represent a superposition of sharp and broad signals)^20,24^.

In summary, the mainly experimental studies of spin-labeled proteins achieved qualitative understanding of ESR spectra. However, exact relationship between the shape of the spectrum and the underlying complex dynamics has remained elusive. In attempt to clarify this relationship, many researchers turned to MD simulations. On a simple level, MD data have been used to characterize the conformational diversity of R1 side chain^25–27^. This proved to be particularly important for DEER experiments, which became a forefront of protein ESR^28^.

In a more ambitious line of approach, MD data were also used to simulate ESR spectra. Initially, short MD trajectories have been employed to construct simplified models for dynamics of R1. For instance, Steinhoff and Hubbell assumed that rotameric jumps of R1 side chain are controlled by a certain simple potential that can be recovered from the MD data^29^. They further assumed that the overall tumbling of the protein can be adequately modeled by means of Brownian dynamics. This description has been used as a basis to generate the so-called stochastic trajectories, which in turn were used to calculate the spectra. Budil and co-workers used MD data to parameterize the motion of nitroxyl label in a form of reorientational diffusion in orienting potential^30^. They subsequently invoked stochastic Liouville equation (SLE) formalism^31^ to calculate ESR spectra. Sezer and Roux in collaboration with Freed used MD data to construct a discrete-state Markov jump model for R1 side-chain dynamics^32^. They employed this model to generate stochastic trajectories and thus simulate the spectra^33^. This approach was later extended by Tyrrell and Oganesyan, who made use of replica-exchange MD simulations^34^.

The significant breakthrough came when Westlund and co-workers demonstrated (for phospholipids) that ESR spectra can be calculated directly from all-atom MD trajectories without any intermediate constructs^35^. This has been accomplished by numerically integrating the Liouville - von Neumann equation for spin density matrix, where the time-dependence of the Hamiltonians was obtained from the MD data. This method was eventually adapted for spin-labeled proteins by Hustedt and co-workers^36^ and by Oganesyan^37^.

When Hubbell and co-workers pioneered the field of protein SDSL ESR, the relationship between ESR lineshapes and the underlying structural/dynamic factors was understood only in broad terms. Many details remained elusive and subject to speculation. Since then a wealth of relevant structural and dynamic information has been obtained by x-ray crystallography and especially NMR spectroscopy, including data on spin-labeled proteins. Furthermore, spin-labeled proteins were successfully modeled by means of Molecular Dynamics and new methods have been devised to calculate SDSL ESR spectra directly from MD trajectories. *These developments pave the way to more accurate, focused picture of SDSL ESR in the context of protein structure and dynamics*. *The objective of this paper is to build such a picture*. We make use of the ESR and other experimental data from seven spin-labeled mutants of the well-known model protein GB1. The origins of the observed lineshapes are elucidated based on MD simulations with net length of 180 μs (two orders of magnitude improvement over the previous benchmark study in this area^37^). We expect that our findings should help to more accurately interpret SDSL ESR spectra of complex and challenging protein systems that are nowadays investigated by means of this technique. We further anticipate that in future such cutting-edge SDSL-ESR studies will involve an MD modeling component and employ various computational strategies such as described in our paper.

## Results

### MD simulations of ESR spectra and comparison with experiments

As a model system for our study, we have chosen B1 domain of streptococcal protein G (GB1). This 56-residue globular protein has been widely used to study protein folding^38^ and to test new structure-solving methods^39,40^. Despite its small size, GB1 has a well-developed hydrophobic core and a stable fold.

**Figure 1:**
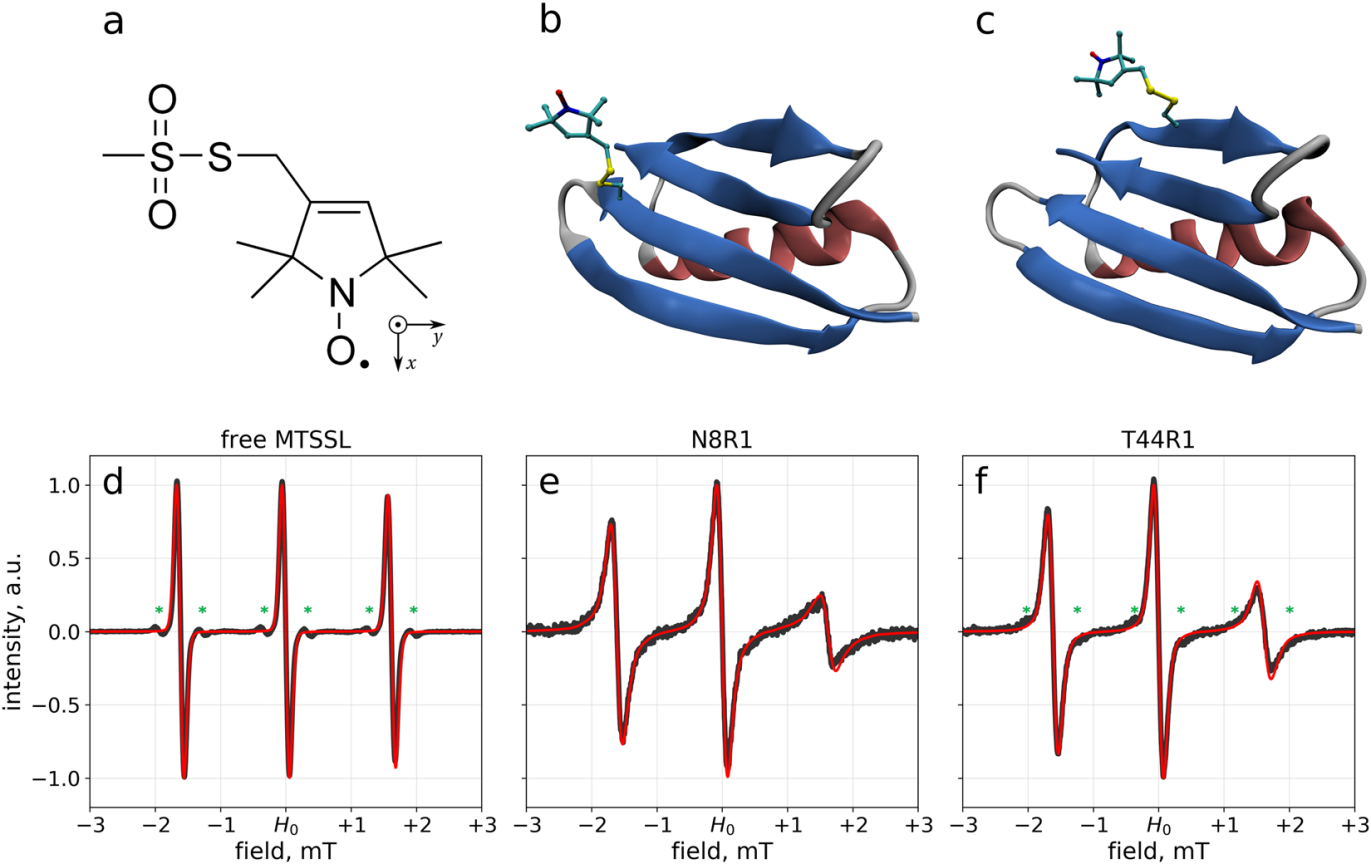
(**a**) Structure of MTSSL molecule and the principal axes system of the hyperfine coupling tensor and g-tensor. (**b,c**) Snapshots from MD trajectories of GB1 N8R1 and T44R1. (**d**) ESR spectrum of free MTSSL (air-equilibrated sample). The spectral features indicated by asterisk are satellites from nuclear spins ^13^C at natural abundance. The experimental and simulated spectra are plotted with broad black line and thin red line, respectively. (**e,f**) ESR spectra of GB1 N8R1 and T44R1. ^13^C satellites are visible at the indicated positions in the T44R1 spectrum. All of the simulated spectra are calculated using Eqs. (1–5), with additional broadening applied to account for the effects of paramagnetic oxygen, inhomogeneous magnetic field and unresolved couplings (the parameters of broadening have been determined experimentally, as detailed in Materials & Methods). Complete summary of spectra from all mutants and trajectories is shown in Fig. S3.

We have considered seven different single-cysteine mutants of GB1: Y3C (buried labeling site), N8C (solvent-exposed site at the middle strand of the β-sheet), K10C (solvent-exposed loop site), E15C and T44C (solvent-exposed sites at the two outer strands of the β-sheet), K28C (solvent-exposed site in α-helix), F30C (buried site). For all of these mutants we prepared structural models with R1 spin label attached to the unique cysteine residue. Specifically, for E15R1 and T44R1 such models were obtained from the crystallographic structures of these particular spin-labeled variants of GB1 (PDB ID 5bmg and 5bmh, respectively). In all other cases, the models were built using the crystallographic structure of the wild-type protein (PDB ID 1pgb) by performing *in silico* mutagenesis and conjugation. Two of these models are illustrated in Fig. 1b,c.

For each of these constructs, the MD trajectory was recorded using Amber16^41^ program equipped with ff14SB^42^ force field in explicit TIP3P^43^ water. The topology and force-field parameters of the R1 tag were as reported previously^44,45^. The simulations were conducted using NPT ensemble at the temperature 293 K. The length of each trajectory was 20 μs. For Y3R1 and K10R1 the simulations were performed in duplicate (the extra trajectories are referred to as Y3R1^#^ and K10R1^#^ trajectories). In addition, we have also recorded 5-μs-long trajectories of free MTSSL, see Fig. 1a, and MTSSL_Δ_ (a product of MTSSL reaction with thiol with subsequent reduction of disulfide bond).

The MD trajectories were used to simulate ESR spectra. As usual, we considered a pair of spins: electron spin $S=1/2$ and ^14^N nuclear spin $I=1$ (corresponding to Hilbert-space matrices 6 × 6). The two prevalent interactions in this system can be represented as follows:

1 $${\bf{H}}(t)={\mu }_{B}{\bf{S}}\cdot {{\bf{g}}}_{iso}\cdot {{\bf{B}}}_{{\bf{0}}}+{\mu }_{B}{\bf{S}}\cdot ({\bf{R}}(t)\cdot {{\bf{g}}}_{aniso}^{PAS}\cdot {\bf{R}}{(t)}^{-1})\cdot {{\bf{B}}}_{{\bf{0}}}+{\bf{S}}\cdot {{\bf{A}}}_{iso}\cdot {\bf{I}}+{\bf{S}}\cdot ({\bf{R}}(t)\cdot {{\bf{A}}}_{aniso}^{PAS}\cdot {\bf{R}}{(t)}^{-1})\cdot {\bf{I}}.$$

Here the Zeeman coupling tensor ${\bf{g}}$ and the hyperfine coupling tensor ${\bf{A}}$ are divided into isotropic and anisotropic part. The latter is expressed in the principal axes system (PAS), which is determined by the local covalent geometry at the radical site. The coordinate frame transformation from PAS to the laboratory frame is represented by the matrix ${\bf{R}}(t)$. This matrix is extracted from the MD coordinates on per-frame basis. The values of ${\bf{g}}$ and ${\bf{A}}$, as well as the definition of PAS, are summarized in the Supporting Information (SI), section 1.

The Hamiltonian ${\bf{H}}(t)$ constructed from the MD data is subsequently used to calculate the evolution of the spin-density matrix:

2 $${\boldsymbol{\sigma }}(\tau )={{\boldsymbol{\Gamma }}}^{-1}(\tau ){\boldsymbol{\sigma }}(0){\boldsymbol{\Gamma }}(\tau ),$$

3 $${\boldsymbol{\Gamma }}(\tau )=\langle \exp (i{\bf{H}}(t)\delta )\exp (i{\bf{H}}(t+\delta )\delta )\cdot \cdot \cdot \exp (i{\bf{H}}(t+\tau )\delta )\rangle .$$

Here the propagator ${\boldsymbol{\Gamma }}(\tau )$ is constructed from the elementary propagators corresponding to the short MD sampling step, $\delta =1\text{}{\rm{p}}{\rm{s}}$, and the angular brackets correspond to averaging over the MD trajectory. The algorithmic implementation of these calculations is described in the SI. The initial state of the system is taken to be ${\boldsymbol{\sigma }}(0)={{\bf{S}}}_{x}$.

Finally, the calculated ${\boldsymbol{\sigma }}(\tau )$ is used to generate the free induction decay:

4 $$FID(\tau )=Tr\{{{\bf{S}}}_{+}{\boldsymbol{\sigma }}(\tau )\}.$$

In turn, ${FID}(\tau )$ is used to calculate the first-derivative ESR spectrum. This is accomplished by means of the special algorithm^46,47^, which emulates the standard detection method used to record continuous-wave (CW) ESR spectra – namely, field modulation, followed by phase sensitive detection at the modulation frequency:

5 $$s(\omega )=-\,\text{Im}({\int }_{0}^{\infty }{FID}(\tau ){J}_{1}({H}_{m}\tau /2)\exp (-i\omega \tau )d\tau ).$$

Here ${J}_{1}(x)$ is Bessel function of the first kind of order 1 and ${H}_{m}$ is the peak-to-peak amplitude of the modulation field. In the calculations, ${H}_{m}$ is set to the same value as used in our experimental measurements, 0.05 mT (prior to being inserted into Eq. (5), it needs to be converted to the units of rad/s).

It is convenient to begin the discussion with the spectrum of free MTSSL. There are several mechanisms that contribute to the spectral linewidth: (i) intrinsic relaxation that stems from Eqs. (1–5); (ii) intermolecular relaxation due to paramagnetic O_2_ contained in the solvent; (iii) broadening due to inhomogeneous static magnetic field; (iv) apparent broadening due to unresolved hyperfine couplings to ^1^H spins in proxyl ring and remote ^13^C spins (at natural abundance). For small rapidly moving molecule such as MTSSL, the mechanisms (i) and (ii) give rise to a Lorentzian lineshape. On the other hand, contributions (iii) and (iv) can be most reasonably approximated by a Gaussian contour. The convolution of these two functions produces the so-called Voigt profile, which has been widely used in fitting of ESR spectra.

We have used this paradigm to analyze two experimental spectra of free MTSSL: one from the air-equilibrated sample and the other from the degassed sample. The fitting procedure used in these analyses is described in detail in the Materials & Methods and illustrated in Fig. S2. For oxygen-induced relaxation, (ii), the determined line broadening is 13.5 μT (full width at half height of the respective Lorentzian contour). For a combination of magnetic field inhomogeneity and unresolved couplings, (iii) and (iv), the determined line broadening is 126 μT (full width at half height of the respective Gaussian contour).

Considering these results, one should bear in mind two important points. First, the obtained parameters are relevant not only for the samples of free MTSSL, but also for the samples of spin-labeled GB1 at solvent-exposed sites. Indeed, the level of oxygen in the air-equilibrated buffer solution, as well as field inhomogeneity specific to our ESR spectrometer and the pattern of unresolved couplings in the proxyl ring, are all invariant factors. In what follows, we use the above results in our predictions of CW ESR spectra from all of the studied samples. Second, we note that mechanisms (ii-iv) dominate the width of spectral lines of free MTSSL, but play a lesser role in the spectra of spin-labeled GB1, which are primarily influenced by the mechanism (i). Therefore, our MD-based predictions of GB1 spectra mainly reflect the calculations based on Eqs. (1–5), whereas the contributions (ii-iv) play a role of a modest constant “offset”.

With this understanding in mind, we undertake the comparison of the experimental and simulated ESR spectra. Figure 1 illustrates the results of this comparison for free MTSSL, as well as N8R1 and T44R1 samples. The two selected labeling sites are located at the inner strand and the outer strand of the same β-sheet in GB1 domain (of note, properties of spin labels attached to sites in β-sheet received far less attention^20^ than those attached to helical sites^2^). While in both cases the R1 side chains are projected into solvent, see Fig. 1b,c, it is expected that N8R1 should experience more interference from neighboring side chains due to its position in the inner strand. Indeed, its spectrum is appreciably broader and more asymmetric than that of T44R1.

We find it convenient to characterize the spectra through two descriptive parameters: width of the central line Δ_(0)_ (defined as frequency separation between the maximum and the minimum of the first-derivative lineshape) and the ratio of amplitudes of the central line and high-field line, ${h}_{(0)}/{h}_{(-1)}$ (where amplitude is defined as intensity difference between the maximum and the minimum of the first-derivative lineshape)^48,49^. The simulated Δ_(0)_ values prove to be in good agreement with the experiment: 169 vs. 166 μT for N8R1 and 149 vs. 150 μT for T44R1. The ${h}_{(0)}/{h}_{(-1)}$ values are in fair agreement: 3.9 vs. 4.3 for N8R1 and 3.0 vs. 3.7 for T44R1. These numbers provide a quantitative check on the visual comparison of the simulated and experimental data, Fig. 1e,f. We conclude that the simulations achieve good accuracy in reproducing the experimental spectra, but fall short of perfect agreement.

One additional comment is in order with regard to the overall tumbling of GB1. It is generally believed that MD simulations fail to correctly capture protein tumbling and reproduce the corresponding correlation time, ${\tau }_{rot}$. This is due to limited length of most trajectories and/or shortcomings of MD models. To deal with this situation, the following strategy has been developed. During the processing of MD data, the overall tumbling is first eliminated and after that reintroduced using the so-called Brownian dynamics approach^36,37^. As input parameters for Brownian dynamics, this method uses the experimentally measured or theoretically predicted components of the rotational diffusion tensor. Thus, the altered trajectory correctly represents the reorientational motion of protein molecule.

In our paper, we do not resort to any such post-processing strategy. Instead, the MD trajectories have been used “as is” to calculate the ESR spectra. The overall tumbling time of spin-labeled GB1 in our simulations was determined to be 3.35 ns (with shortest ${\tau }_{rot}$ of 3.22 ns found in F30R1 and longest ${\tau }_{rot}$ of 3.44 ns found in Y3R1^#^, simulation temperature 293 K). These results are in reasonable agreement with the experimental findings, 4.1 ns (after correction for 10% D_2_O)^50,51^, as well as theoretical predictions, 3.9 ns^52^. Furthermore, our approach automatically takes care of tumbling anisotropy, which is significant for the GB1 domain, ${D}_{\parallel }^{rot}/{D}_{\perp }^{rot}=1.39-1.45$^51^. Additional details, including the discussion of solvent viscosity in our MD model, are given in the section 2.1 of the SI.

### Placing spin label into buried sites

We have made special effort to engineer variants of GB1 where spin label is positioned in the interior of the protein and thus is sterically constrained. Initially, four potential mutation sites have been considered: Y3, F30, W43 and F52. These are all bulky residues and, therefore, we could reasonably hope that R1 can be accommodated at these sites. The original level of solvent exposure for all of these residues is low (according to crystal coordinates, 2–3%, except for W43 where it reaches 23%). As a first step, we recorded short (200 ns) MD trajectories for Y3R1, F30R1, W43R1 and F52R1. The first two constructs showed the lowest backbone *rmsd* and the fewest R1 rotameric jumps during these MD simulations. Accordingly, they have been selected for further experimental and computational studies.

For both Y3R1 and F30R1 constructs, the experimental and simulated (based on 20-μs MD trajectory) spectra appear to be in good agreement, see Fig. S3a,b,h. For both mutants the spectra are substantially broadened, as can be expected for sterically confined R1 side chain. At the same time, the experimental spectra display a sharp feature, which is clearly visible against the background of the broadened high-field line (indicated by dagger in the plots). This sharp feature is not reproduced in our simulations. This observation prompted us to take a closer look at Y3R1 and F30R1 samples.

For this purpose, we have prepared the recombinant ^15^N-enriched sample of GB1 F30C and recorded an HSQC spectrum of this sample. Subsequently, we labeled this sample with MTSSL, reduced the paramagnetic label by applying ascorbate, and then recorded another HSQC spectrum. The superposition of the two spectra, from F30C and F30R1 (diamagnetic) is shown in Fig. S4. The changes in the spectral map due to spin labeling are rather profound. First, almost all of the peaks are shifted away from their original positions. Second, many peaks are strongly attenuated/broadened. This is particularly true of the resonances corresponding to the secondary-structure elements, whereas the peaks corresponding to flexible segments at around 8.3 ppm ^1^H chemical shift retain their original intensity (summarized in Fig. S5). It is clear that F30R1 spectrum suffers from extensive broadening, which stems from an exchange process on μs-ms time scale possibly accompanied by oligomerization. It is also apparent that this process has global rather than local character.

Of note, it has been previously shown that mutations in the core of GB1 can trigger global conformational exchange on millisecond time scale^53^. Furthermore, a number of GB1 mutants have been reported that lose some of the secondary structure, but regain stability by forming dimers, tetramers or higher-order assemblies^54–56^. One such example is a completely intertwined tetramer, featuring a number of long flexible loops^57^. We argue that incorporation of a fairly bulky R1 tag into the hydrophobic core of GB1 may lead to similar detrimental effects. In particular, partial unfolding of GB1 and concurrent formation of oligomers would greatly complicate any attempt to obtain a quantitative interpretation of the ESR spectra.

To test this possibility we have manufactured three different samples of GB1 F30R1. The first one was labeled in a standard fashion by incubation with MTSSL; the unreacted MTSSL was removed using a desalting column (see Materials & Methods). The second one was subjected to additional purification step using ion-exchange chromatography. The third one was labeled in the presence of 8 M urea with the intent to provide ready access to the otherwise poorly accessible labeling site. The three resulting spectra are clearly different, see Fig. S6. The labeling procedure using urea led to a sharp spectrum, suggesting that F30R1 fails to refold following the transfer to refolding buffer^58^. The other two samples gave rise to multicomponent spectra with different degree of broadening, thus suggesting that spin-labeled material consists of a mixture of species that, in principle, can be sorted using various separation techniques. Similar behavior has been observed for GB1 Y3R1 (not shown).

Next, we turn to the discussion of MD trajectories of F30R1 and Y3R1. As it turns out, these trajectories do not show any unusual behavior. The *rmsd* trace of both constructs show that they remain structurally invariant during the 20-μs simulations, see Fig. S7a,b,h. For F30R1, the secondary-structure Cα *rmsd* relative to the crystallographic coordinates is mostly around 0.6 Å, same as for the samples with surface labeling sites. For Y3R1, one of the trajectories shows evidence of a long-lived state with *rmsd* of 2.4 Å, but eventually it morphs into the more familiar form with *rmsd* of ca. 1.3 Å. Thus, MD simulations show no evidence of major structural instability that is seen in our NMR and ESR experiments. There is no contradiction in this outcome, however. Indeed, our 20-μs trajectories are not long enough to capture major structural rearrangements such as inferred from our experimental observations. For example, longer simulations have been needed to observe thermal unfolding of small globular proteins^59,60^. Note also that ^15^N line broadening in the HSQC spectrum of F30R1 (diamagnetic) is consistent with exchange process on the scale of hundreds of microseconds to several milliseconds. This time scale exceeds the length of our MD trajectories.

In summary, inserting R1 tag into the interior of GB1 causes major changes in the status of the sample. In particular, partially or fully unfolded species emerge, as evidenced by the ESR spectra. In turn, this brings about the possibility of self-association and formation of misfolded oligomeric species. We believe that such unfavorable scenario is not uncommon – any attempt to incorporate spin label into protein’s hydrophobic core may be fraught with such complications. This is similar to the effect of destabilizing mutations^61,62^, but perhaps more pronounced due to the relatively large size of the R1 tag. For these potentially misbehaving samples, it is difficult or impossible to build a faithful MD model. Conversely, simple MD models (such as ours) can lead to a gross misinterpretation of ESR spectra. Therefore, despite the apparent similarity of the experimental and simulated spectra of Y3R1 and F30R1, we abstain from making any claims in this regard. The observed reasonably good agreement may or may not reflect the reality.

One should reckon with a possibility that such behavior may also be encountered in other systems. Incorporation of R1 tag sometimes has a destabilizing effect on protein structure, giving rise to a fraction of species that are partially or fully unfolded. In turn, these species are often prone to aggregation. The resulting complex mixture can produce a multicomponent ESR spectrum, containing both broad and sharp signals, as documented previously^63–65^.

### Simple determinants of the ESR spectral shapes

The results in Fig. 1 demonstrate that our computational methodology is successful in reproducing the experimental ESR spectra of spin-labeled GB1 with R1 tag attached to the sites on the protein surface. In principle, MD-based calculations can provide unparalleled sophistication and accuracy in predicting the ESR spectra (limited only by the accuracy of the force field and the length of the MD simulations). However, at the same time we would like to obtain simpler, more qualitative insights into the origins of the ESR lineshapes. This can be accomplished by analyzing the MD trajectories in conjunction with the simulated spectra.

In the previous section, we discussed the constructs where R1 tag is attached at the buried sites, Y3R1 and F30R1. In reality, these samples are probably more structurally diverse than it appears from our limited-length trajectories. Nevertheless, these two pieces of data are formally valid in a sense that the underlying MD trajectories are consistent with the simulated spectra. Specifically, these trajectories represent a (desirable) situation where the insertion of R1 into the protein interior causes only minor structural perturbations. So long as our discussion is limited to the MD-based results (notwithstanding the experimental data), these two trajectories can be included in the analysis alongside with others.

**Figure 2:**
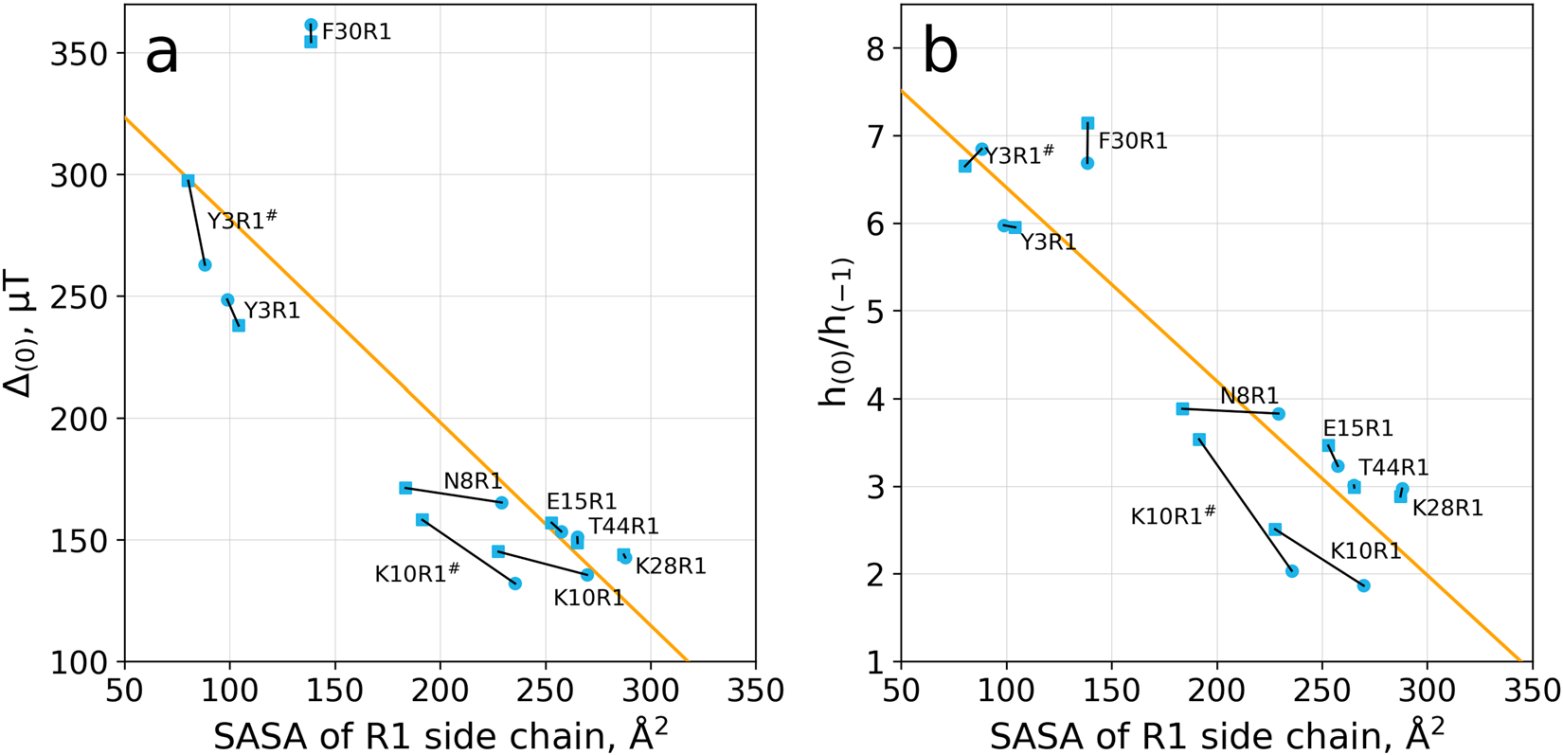
Correlation between spectral shape descriptors Δ_(0)_, ${h}_{(0)}/{h}_{(-1)}$ and solvent-accessible surface area of R1 side chain according to the MD data and MD-based spectral simulations. To give a better idea of convergence properties, we have divided each 20-μs trajectory into two halves and processed them separately. The results from this procedure are shown in the plot by the pairs of symbols connected by line segments (circle represents the first half of the trajectory and square the second half).

As discussed in the Introduction, the shape of ESR spectra is largely determined by conformational dynamics of the R1 tag. In turn, the dynamics of R1 depends on its environment – it can be highly mobile when surrounded by water or otherwise completely immobilized when constrained by the elements of protein structure. To validate this concept, we have calculated the average solvent-accessible surface area (SASA) of R1 side chain in each of our MD simulations. Next we correlated the obtained SASA values with the spectral shape descriptors: width of the central line Δ_(0)_ and asymmetry quotient ${h}_{(0)}/{h}_{(-1)}$. The results are presented in Fig. 2.

A good correlation is observed between the solvent-accessible surface area of R1 side chain and the two principal characteristics of the ESR spectra: line broadening and asymmetry. Thus, our MD simulations confirm the existing notion that the interaction of R1 tag with the protein matrix (as opposed to solvent) is the main factor that determines the shape of the spectrum^2,66,67^. Interestingly, however, the detailed examination of the results in Fig. 2 suggest that there are other, more subtle factors in play. There are examples where two halves of the trajectory show near identical SASA, but appreciably different spectral characteristics. In other cases, appreciably different SASA values correspond to near identical spectral parameters. The origins of this behavior are discussed in what follows.

Beyond the degree of burial, it has been suggested that ESR lineshapes are also sensitive to local backbone dynamics. In general, such sensitivity can be expected of all solvent-exposed surface sites where R1 tag has little interaction with other side-chain or backbone groups^48^, but specific examples mainly involve α-helices^2,67,68^. Our simulations provide a good opportunity to explore the role of backbone dynamics and assess its influence on the ESR lineshape. For this purpose, we have selected the trajectory K28R1, where the labeling site is in α-helix and R1 tag is projected into solvent. To visualize the relevant motional modes we have plotted temporal auto-correlation functions representative of (*i*) full dynamics of the NO bond from the R1 proxyl ring, i.e. the *x* axis of PAS, (*ii*) full dynamics of the vector perpendicular to the R1 proxyl ring, i.e. the *z* axis of PAS, (*iii*) overall protein tumbling, and (*iv*) local dynamics of the C^α^C^β^ bond from R1 residue in the molecular frame of reference (see Fig. S8g).

The inspection of the results demonstrates that there is very little backbone motion in the sole α-helix of GB1. The amplitude of local backbone dynamics at this site is small, corresponding to the order parameter ${S}_{C\alpha C\beta }^{2}=0.93$, cf. green curve in Fig. S8g. This observation is confirmed by the NMR relaxation study by Sheppard *et al*. that reports ${S}_{C\alpha C\beta }^{2}=0.92$ for residue K28 in GB1^51^. It is also consistent with dynamics analyses in the closely related protein GB3 using residual dipolar coupling data^69^. Consequently, local backbone dynamics makes only minor contribution to the dynamics of the spin label, green curve vs. orange and magenta curves in Fig. S8g.

We observe that this situation is typical for well-folded globular proteins: their scaffold consisting of α-helices and β-sheets is usually rather rigid, as demonstrated by numerous NMR studies^70,71^. When such proteins are spin-labeled at α-helical or β-sheet sites, small-amplitude backbone motions should have only marginal effect on the ESR lineshape. Furthermore, this effect is masked by other factors – primarily, by dynamics of R1 side chain, which is in turn influenced by its site-specific environment. In this situation, the usefulness of the SDSL method for studies of backbone dynamics is necessarily limited.

### Specific interactions involving R1 tag

While the degree of solvent exposure is a good predictor of ESR lineshape, it is also rather crude. Even those R1 tags that are located on the protein surface can make certain specific contacts with the surrounding protein sites. In particular, it has been suggested that R1 can engage in hydrogen bonds or electrostatic interactions with the surrounding protein groups, or otherwise pack against hydrophobic patches on the protein surface^20,21^. Furthermore, it has been envisioned that R1 tag can jump between different rotameric states, where each state is characterized by a distinct set of interactions with the proximal protein sites. In particular, it can happen that nitroxide label is largely immobilized in one of the states, but moves freely in the other. If the dynamic exchange between such two states happens to be slow (compared to the decay of the electron spin $FID$), such system should give rise to a distinctive multicomponent spectrum^24,64^.

In our MD simulations, we have indeed observed the examples of R1 tag sampling different environments. Consider, for instance, the trajectory K10R1^#^, where the tag is attached to the loop *L1* (see Fig. S9 for loop nomenclature)^72^. The simulation starts from the structure where R1 is extended into solvent, pointing away from the body of the protein, see Fig. S9a. This configuration, where R1 has considerable motional freedom, exists until ca. 3.3 μs. Hereafter, we refer to it as state A.

After that, the conformation of the loop *L1* begins to change. Its initial β-turn topology is disrupted. The side chain of the C-terminal residue E56, which originally bridges loops *L1* and *L3*, moves away. Eventually, at ca. 5.0 μs a new arrangement is formed, where R1 makes extensive contacts with the side chains of residues L12, Y33, N37, V39 and L7 (listed here in the order from most important to least important). In the crystal structure, all of these side chains belong to the exposed edge of the protein core, which is sandwiched between the four-strand β-sheet and the sole α-helix of GB1. The described arrangement exists (with one relatively short interruption) until ca. 17.9 μs. It is characterized by reduced mobility of the proxyl ring, which is directly involved in the above contacts (illustrated in Fig. S9b). We refer to it as state B.

Finally, toward the very end of the trajectory, the *L1* loop bends backward and the R1 tag makes contacts with several residues on the outer side of the β-sheet: T44, I6, T53 and even D46 and T49 (illustrated in Fig. S9c). This latter state is relatively short-lived, ca. 1 μs. We refer to it as state C.

Altogether, the trajectory K10R1^#^ demonstrates an impressive diversity of different states sampled by the spin label. This diversity explains the lack of convergence in the results from K10R1^#^ in Fig. 2. Specifically, the second half of the trajectory involves a greater proportion of state B, where the proxyl ring becomes associated with the surface of the protein and shows reduced mobility. The dominant role in the formation of state B belongs to van der Waals packing of the proxyl ring with the mainly hydrophobic side chains, such as L12 and Y33. This is accompanied by a sharp reduction in R1 solvent accessible surface area – SASA is decreased by 200 Å^2^ in going from state A to state B (more than 2-fold drop). In contrast, the role of hydrogen bonds appears to be minimal. We have found that R1 tag forms (conventionally defined) hydrogen bonds in only 2.7% of all frames in the trajectory.

It is worth noting that analysis of K10R1^#^ trajectory can also be framed in terms of R1 burial, similar to what has been described in the previous section. However, in this particular case we distinguish several states, each with its own characteristic SASA. It is also important which particular portion of the R1 tag is buried. It is clear that packing of the proxyl ring against the body of the protein can lead to significant line broadening. On the other hand, confinement of S^γ^ atom leads only to partial restriction of the R1 tag and, therefore, should have less effect on the spectrum. Other fine details of R1 dynamics, which are not necessarily reflected in SASA changes, can also influence the lineshapes.

Finally, the spectrum calculated from the trajectory K10R1^#^ should be viewed as a multicomponent spectrum. It is comprised of several components, corresponding to the states A, B and C that exist in slow exchange with each other. Although the dynamic properties of these states are significantly different, they are not dramatically different. Therefore, visual inspection of the K10R1^#^ spectrum fails to detect its multicomponent character, see Fig. S3e.

States that are similar to A and B are also observed in the trajectory K10R1 along with one other state, which has no direct equivalent in K10R1^#^. Distinct states with sufficiently long lifetimes (several μs) are also observed in other trajectories, e.g. N8R1. In all these cases, the simulated spectra cannot be easily identified as multicomponent spectra, see Fig. S3. On the other hand, those spectra that are visually identified as multicomponent spectra may often result from partial protein unfolding, cf. the preceding discussion.

### Conformational dynamics of solvent-exposed R1 tag

**Figure 3:**
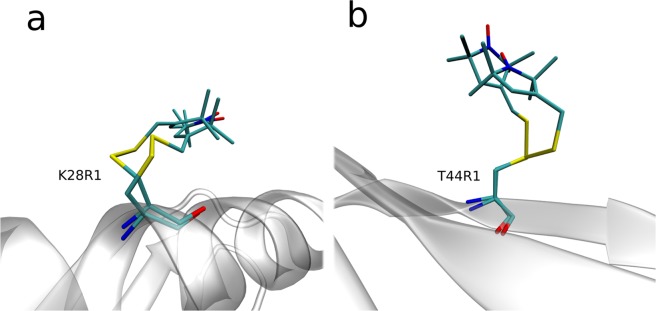
Solvent-immersed R1 tag before and after conformational transition (i.e. concerted jump in multiple torsional angles ${\chi }_{i}$) as seen in the MD simulations of spin-labeled GB1. The images represent two closely spaced (time separation 3 ps) MD frames corresponding to: (**A**) the moment in time 0.46 μs in the trajectory K28R1 and (**B**) the moment in time 15.3 μs in the trajectory T44R1. Prior to making the plot, the two structures are superimposed via all C^α^ atoms belonging to the secondary-structure regions; the cartoon representation of the protein backbone corresponds to the first of the two frames. Of note, graph (**A**) illustrates the transition between the rotameric states that are most typical of α-helical labeling sites, $({\chi }_{1},{\chi }_{2})=(m,m)$ and $(t,p)$, whereas graph (**B**) illustrates the transition between the states that are most typical of β-sheet sites, $({\chi }_{1},{\chi }_{2})=(t,m)$ and $(t,t)$. For ${\chi }_{1}$, ${\chi }_{2}$ and ${\chi }_{4}$ rotamers we use the *t*, *p*, *m* nomenclature by Lovell *et al*.^125^, while for ${\chi }_{3}$ and ${\chi }_{5}$ rotamers we use the analogous *p*, *m* nomenclature (in the latter case the torsional angle is defined by the doubly bonded carbon atom in the proxyl ring).

Hubbell and co-workers have found that solvent-exposed R1 tag on a surface of an α-helix has certain distinctive conformational preferences. Specifically, it favors conformational states $({\chi }_{1},{\chi }_{2})=(m,m)$ or $(t,p)$ (see caption of Fig. 3 for discussion of notations). This finding is based on a significant number of crystallographic structures solved in the Hubbell’s laboratory, as well as other groups^73–75^. As it turns out, in both of these rotameric states S^δ^ atom is positioned in close proximity to the C^α^-H^α^ group of R1. Initially, it has been suggested that this arrangement corresponds to an unconventional hydrogen bond^68^. Later, the same putative interaction was attributed to a favorable van der Waals contact^76^. It was proposed that this interaction is responsible for the propensity of the R1 side chain to adopt either $(m,m)$ or $(t,p)$ conformations. It has been further suggested that ${\chi }_{1},{\chi }_{2}$ and ${\chi }_{3}$ dynamics are slow and, therefore, have little direct impact on the shape of the spectrum^68^. Consequently, the relevant R1 motions should be largely limited to ${\chi }_{4}$ and ${\chi }_{5}$ jumps. This scenario became known as “X_4_/X_5_ model”^48^.

While spin labels with attachment sites in α-helices have been investigated quite extensively, relatively little has been done on spin labels with attachment sites in β-sheets. The currently available crystallographic evidence includes 37 realizations of solvent-facing R1 tags in the β-sheet regions, including our own previous work on GB1^77–81^. The sample is dominated by $({\chi }_{1},{\chi }_{2})=(t,m)$ and $(t,t)$ species (41% and 27%, respectively). Both of these forms do not show the putative interaction S^δ^···C^α^-H^α^.

Based on this evidence, we suggest that the preferred rotameric states $({\chi }_{1},{\chi }_{2})$ of the solvent-exposed R1 tag are mainly determined by the backbone topology. Specifically, in α-helices the favored conformers are $(m,m)$ and $(t,p)$, while in β-sheet they are $(t,m)$ and $(t,t)$. The proximity of S^δ^ and C^α^-H^α^ atoms is likely a consequence rather than the root cause of the conformational preferences observed in α-helices. The stabilizing effect of S^δ^···C^α^-H^α^ interaction on $(m,m)$ and $(t,p)$ conformers appears to be more limited than initially thought, although it probably has certain influence along with several other subtle interactions^48^.

Next, we consider the other part of the X_4_/X_5_ model – namely, the assumption that ${\chi }_{1},{\chi }_{2}$ dynamics is slow. To glean some information about torsional-angle dynamics in R1 tag attached to the surface of α-helix we turn to the MD trajectory K28R1. To assess the characteristic time scale of ${\chi }_{i}$ dynamics, we have computed the temporal correlation functions ${\gamma }_{i}(\tau )=\langle \cos \,\Delta {\chi }_{i}(\tau )\rangle$, see Fig. S10a. From this graph it can be seen that the lifetime of ${\chi }_{1}$ and ${\chi }_{2}$ rotamers is on the order of several nanoseconds. This result is consistent with the known magnitude of rotational barriers in alkanes, 3–5 kcal/mol^82^, and more specifically, in the R1 residue^76^. It is also consistent with MD/NMR data on side-chain dynamics^83^. We conclude that for solvent-facing R1 tag on the surface of α-helix the rotameric jumps involving ${\chi }_{1}$ and ${\chi }_{2}$ should not necessarily be viewed as a slow form of motion.

Further analysis of the data in Fig. S10a shows that ${\chi }_{4}$ and especially ${\chi }_{5}$ dynamics is faster than ${\chi }_{1},{\chi }_{2}$ dynamics, on the order of hundreds of picoseconds. At the same time, ${\chi }_{3}$ dynamics is substantially slower, on the order of 100 ns. This latter result is also expectable and consistent with the known magnitude of the barrier to rotation about the SS bond, ca. 7 kcal/mol^84^. All of the above observations also hold for the labeling sites in the β-sheet, Fig. S10b–d, although the details obviously differ from one site to another.

Summarizing the above discussion, in the solvent-immersed R1 tag ${\chi }_{1}$ and ${\chi }_{2}$ jumps can have a direct impact on the ESR lineshape (while ${\chi }_{3}$ dynamics has only indirect influence, viz. it may influence the local environment of the spin label as discussed in the previous section). Yet, our further observations suggest that the impact of ${\chi }_{1},{\chi }_{2}$ jumps on the spectral lineshape is less than may be expected. This is because ${\chi }_{i}$ jumps occur in a concerted manner and largely compensate each other with respect to the motion of the paramagnetic center.

The situation is illustrated in Fig. 3a, which shows R1 conformations from the two closely spaced MD frames in the trajectory K28R1. The time separation between these two frames is 3 ps. During this short time interval, the following ${\chi }_{i}$ transitions take place: ${\chi }_{1}$ from rotamer *m* to rotamer *t*, ${\chi }_{2}$ from *m* to *p* and ${\chi }_{5}$ from *p* to *m*. All of these jumps occur in a concerted manner such that the resulting displacement/reorientation of the proxyl ring turns out to be rather minimal. Specifically, the orientation of the NO bond, which is relevant for the evolution of the spin-density matrix ${\boldsymbol{\sigma }}(t)$ and the ESR lineshape, changes only by 23° as a result of this particular conformational rearrangement, Fig. 3a. Over the same time interval, the orientation of the normal to the proxyl ring changes by 20°. This is much less than could be (naively) expected given the magnitude of changes in the individual torsional angles.

The above compensation effect is rather simple to rationalize. The proxyl ring at the end of the R1 tag is relatively bulky and experiences a substantial hydrodynamic drag. On the other hand, the portion of R1 tag extending from C^α^ to C^ζ^ can be viewed as a segment of polymer chain, which is sufficiently flexible. For the solvent-immersed tag, the conformation of the C^α^-C^ζ^ “linker” can change rather extensively, while the heavy proxyl ring remains relatively immobile. Consequently, the effect of rotameric jumps on the ESR lineshape is less than can otherwise be expected. The situation is similar to the one that has been encountered in the NMR/MD studies of naturally occurring side chains on a protein surface^85^.

Similar conclusions can be drawn about the concerted transitions in R1 tags attached to β-strands. This is illustrated in Fig. 3b. In this case, ${\chi }_{2}$ converts from rotamer *m* to rotamer *t*, ${\chi }_{4}$ from *p* to *t* and ${\chi }_{5}$ from *p* to *m*. The resulting displacement of the proxyl ring (2.0 Å) and the change in orientation of the NO bond (33°) are relatively modest. Once again, there is a self-compensatory effect in a sense that multiple ${\chi }_{i}$ jumps cause little movement of the proxyl ring. It should be noted, however, that a comprehensive analysis of correlated motions in R1 tag could be complicated. Such analysis should address both rotameric jumps and smaller angle adjustments. Furthermore, it should discriminate between productive transitions and short-lived fluctuations. In addition, it should deal with the time lag between the correlated changes in different torsional angles. This problem requires special mathematical approaches^86,87^ and awaits further investigation.

In summary, the above results offer a new perspective on the X_4_/X_5_ model^68^. The original model posits that the ESR lineshapes are mainly affected by jumps in the ${\chi }_{4},{\chi }_{5}$ angles and that ${\chi }_{1},{\chi }_{2},{\chi }_{3}$ jumps are slow. We find that ${\chi }_{1}$ and ${\chi }_{2}$ jumps can be sufficiently fast to have direct influence on the shape of the ESR spectrum. However, this influence is mitigated by the self-compensatory nature of the torsional-angle dynamics in the R1 tag. Of note, our observations are not limited to samples with labeling sites in α-helices, but apply to all surface sites.

### The role of protein tumbling

Reorientation of GB1 molecule as a whole has a significant influence on the shape of the ESR spectra. For those samples where R1 tag is buried and highly constrained, such as F30R1, this is a dominant form of motion (see Fig. S8h), which dictates the shape of the spectrum. For other samples where R1 is projected into solvent, such as T44R1, the overall tumbling is less important than the conformational dynamics of the tag. Yet it remains a significant factor (see Fig. S8i).

MD simulations offer an attractive option to elucidate the effect of tumbling on the shape of the spectra. During the processing of the MD trajectory it is possible (and, in fact, straightforward) to “switch off” the overall tumbling. This can be accomplished by superimposing protein coordinates from the individual MD frames onto a reference structure. It is also possible to adjust the rate of the protein tumbling, thus effectively altering the protein size. For this purpose, we have developed a special algorithm that is described in the SI, section 2.2. In brief, we extract the rotation matrices corresponding to reorientation of GB1 at each 1-ps step in the trajectory. Then we change the amplitude of these elementary rotations by using the scaling factor $\lambda$ ($\lambda  < 1$ slows down the tumbling, while $\lambda  > 1$ speeds it up). The redefined rotations are used to assemble the so-called pseudo-trajectory, which differs from the original trajectory in only one respect – increased or decreased tumbling rate. Finally, this pseudo-trajectory is used to generate an ESR spectrum using the same direct propagation approach as described above.

The implementation of this algorithm is more challenging than it may appear at a first glance. Specifically, the procedure needs to be designed such as to preserve the correct description of tumbling anisotropy, which is particularly relevant for GB1^51^. To obtain valid results, the elementary rotations need to be defined in the molecular frame of reference (see SI section 2.2 for details).

We have applied this approach to all 20-μs trajectories recorded in our study using *λ* = 0.5 and *λ* = 2.0. This corresponds to a 4-fold increase/decrease in the protein tumbling correlation time ${\tau }_{rot}$. The results from these calculations are shown in Fig. S11. For the mutants with buried R1 tag, such as F30R1, the effect of changes in the tumbling rate is quite dramatic. This can be readily understood because the spectral shape in this case depends almost entirely on the tumbling rate. In the slow tumbling regime, *λ* = 0.5, the spectrum (blue curve in Fig. S11h) resembles those that have been observed under slow-motion conditions^3^. On the contrary, the constructs where R1 tag is projected into solvent, such as T44R1, are not as sensitive to changes in the tumbling rate. In particular, the slowing of the overall tumbling for T44R1 has only limited effect on the spectrum (cf. red and blue curves in Fig. S11i). This can also be readily rationalized given that the motion of the paramagnetic center and the resulting “orientational memory loss” are controlled in this case by the R1 internal dynamics, which is fast compared to the overall tumbling (see Fig. S8i). Therefore, further slowing of the overall tumbling in such sample is largely inconsequential, see Fig. S12i.

We conclude that the method presented in this section can be useful in assessing the relative importance of R1 mobility vs. the overall protein tumbling. This can be relevant for those experimental applications that rely on changes in R1 mobility to register various molecular events. In particular, the results can help to make a decision about use of viscogens in preparing of protein ESR samples, which is a popular experimental strategy to minimize the effect of tumbling on the ESR spectra. The described procedure should also be helpful in a situation where there is a significant difference between the MD-predicted and experimentally determined values of protein tumbling correlation time ${\tau }_{rot}$. Using the above approach, this issue can be easily corrected.

Our method appears to be equivalent to the one that has been developed by Andersen and LeMaster for prediction of NMR relaxation rates^88^. It should be noted, however, that in the context of NMR relaxation the problem of under- or overestimated ${\tau }_{rot}$ can usually be corrected by simpler means^89^. No such simple solutions are available for MD-based calculations of protein ESR lineshapes, which rely on the direct propagation scheme. The described rescaling algorithm offers an answer to this problem.

### Redfield-theory description of the ESR spectra. Cross-correlation (TROSY) effect

An interesting new perspective can be obtained by using MTSSL reagent enriched in ^15^N, which is readily available commercially. As can be expected, the experimental procedure is identical to the one that is used with unlabeled MTSSL (with certain caveats, see Materials & Methods). The computational procedure is also analogous except that a smaller basis is needed to accommodate ^15^N spin 1/2 than ^14^N spin 1.

**Figure 4:**
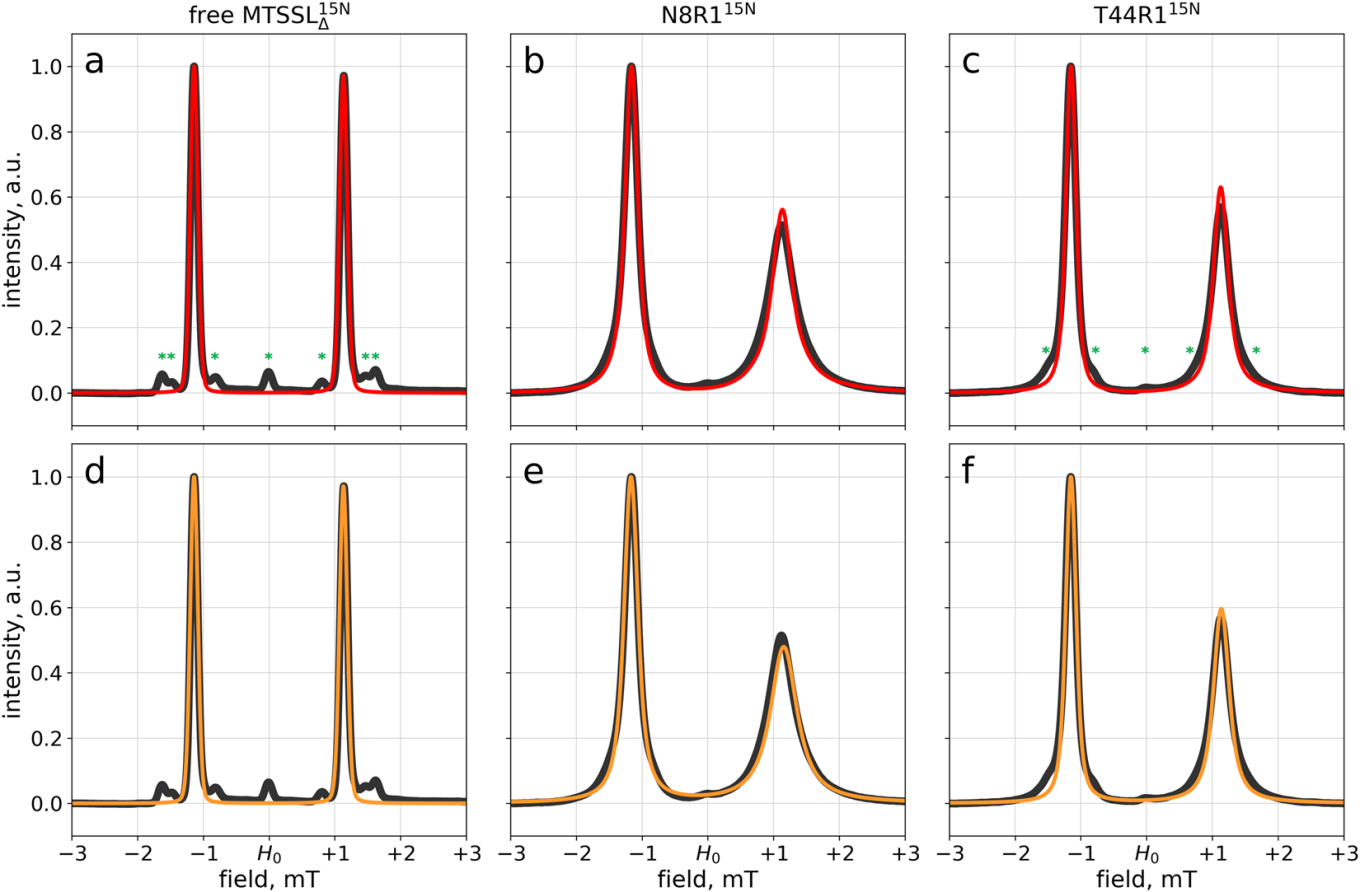
Absorption-mode ESR spectra of free ${{\rm{MTSSL}}}_{\Delta }^{{\rm{15N}}}$, N8R1^15N^ and T44R1^15N^ as obtained from: experimental measurements (black lines), MD-based calculations using the direct propagation scheme (red lines) and MD-based calculations using Redfield formalism (orange lines). The samples are identified in the captions above the plot; the experimental spectra in panels (**d–f**) are the same as in panels (**a–c**). Free ${{\rm{MTSSL}}}_{\Delta }^{{\rm{15N}}}$ corresponds to ^15^N-enriched MTSSL with removed methylsulfonyl group, i.e. ^15^N-(1-oxyl-2,2,5,5-tetramethylpyrroline-3-methyl)-methanethiol. Minor peaks marked by green asterisks are discussed in the text.

We have prepared the samples of N8R1^15N^ and T44R1^15N^, as well as ^15^N-enriched free spin label ${{\rm{MTSSL}}}_{\Delta }^{{\rm{15N}}}$, which have been further used to record ESR spectra. The selection of samples here mirrors that in Fig. 1. However, now we choose to present the spectra in the absorption mode (which allows us to draw an interesting parallel to NMR spectroscopy, see below). For this purpose, we have integrated the experimental first-derivative spectra, as well as the corresponding theoretical spectra obtained by means of the direct propagation scheme, Eqs. (1–5). The results are shown in Fig. 4a–c, where the experimental spectra are plotted as black lines and MD-based predictions as thin red lines.

The spectra in Fig. 4 are doublets, which is expectable: the ESR signal is split into two components corresponding to +1/2 and −1/2 projections of the nuclear ^15^N spin. Similar to Fig. 1, we observe good agreement between the experimental spectra (black lines) and the results of the MD-based calculations using direct propagation scheme (red lines). The spectra also contain minor signals, which are especially clearly seen in Fig. 4a (marked by green asterisks in the plot). The origin of these signals can be readily understood. First, there is a triplet that stems from a small proportion of ^14^N MTSSL in the labeling reagent. Second, there is a pair of doublets corresponding to ^13^C satellites of ${{\rm{MTSSL}}}_{\Delta }^{{\rm{15N}}}$ lines. While all of these minor signals are clearly visible in the spectrum of free label, they are broadened in the spectra of R1-tagged GB1 and strongly overlap with major peaks, see Fig. 4b,c. Yet these minor signals are still identifiable – especially in the spectrum of T44R1^15N^, which is relatively sharp (the corresponding spectral features are indicated by asterisks in Fig. 4c). The minor signals are a part of the experimental spectra, but they have not been included into our MD-based computational scheme (although straightforward, such extension would make the algorithm more cumbersome). Consequently, these minor signals are perceived as small deviations between the experimental and predicted lineshapes (cf. Fig. 4c). However, aside from these small deviations, the agreement between the predictions and the experiment is very good.

The spectra shown in Fig. 4 are reminiscent of asymmetric doublets, which have been thoroughly investigated in the context of heteronuclear NMR spectroscopy^90,91^ and became especially prominent with the advent of TROSY experiments^92^. One line in the doublet is sharp and tall (the so-called TROSY component), while the other is broad and short (anti-TROSY component). Is this a coincidental similarity, or can it be traced to the same underlying spin dynamics mechanism? In order to address this question, we decided to implement an alternative approach to calculation of ESR spectra: namely, use Redfield formalism instead of the direct propagation scheme^93,94^.

In general, our Redfield-theory computational scheme is similar to the one that is widely used in MD-based calculations of NMR observables^95^. Briefly, Redfield matrix is computed for the system at hand using the time-dependent portion of the Hamiltonian Eq. (1). The relevant temporal correlation functions $g(\tau )$ are extracted from the MD data and then converted to spectral densities. Finally, Redfield equation is solved using the standard diagonalization technique to generate an observable ESR spectrum. While this scheme follows the general prescriptions of the Redfield theory, its implementation for the system at hand requires some care. For example, the block-diagonal structure of the Redfield matrix (Redfield kite)^96^ is different in this case from the usual form that is familiar to NMR practitioners. The complete description of our algorithm can be found in the SI, section 3.

The results of our Redfield-theory calculations are illustrated in Fig. 4d–f (orange curves). The agreement with the experiment is clearly very good, on par with the previous results using the direct propagation scheme, Fig. 4a–c. To obtain a better idea about the applicability of Redfield theory, we calculated the standard first-derivative spectra for all available GB1 trajectories and compared them with the prior direct propagation predictions. The results are summarized in Fig. S13.

For Redfield theory to be valid the following condition needs to be fulfilled: ${\tau }_{c}\ll {T}_{2e}$, where ${T}_{2e}$ is the characteristic decay time of transverse electron spin magnetization and ${\tau }_{c}$ is the characteristic decay time of the relevant correlation function. This requirement is readily met for samples with solvent-exposed R1 tag, such as T44R1. These samples are characterized by ${\tau }_{c}$ on the order of 1 ns and ${T}_{2e}$ approaching 100 ns (as determined from $g(\tau )$ and $FID(\tau )$, respectively). Accordingly, in this case Redfield theory produces the results in excellent agreement with the exact calculations, see Fig. S13f,g,i.

The situation is more challenging for those samples where R1 tag is trapped in the protein hydrophobic core, such as Y3R1 and F30R1. In this case, the motion of R1 is mainly due to the overall protein tumbling and the corresponding correlation time ${\tau }_{c}$ approaches ${\tau }_{rot}=3.2\,{\rm{ns}}$ (cf. Fig. S8). Concurrently, the electron spin relaxation time in these samples becomes relatively short, ${T}_{2e} \sim 15-20\,{\rm{ns}}$. Hence, the validity condition of the Redfield theory is pushed to its limits. Consequently, there are appreciable deviations between the predictions of the Redfield theory and the exact results obtained via the direct propagation scheme, cf. Fig. S13a,b,h. Nevertheless, even in this situation Redfield theory provides a fairly accurate approximation of the exact spectra.

Returning to the results in Fig. 4, we reiterate that Redfield theory holds nicely for the two GB1 spectra shown in this graph. This means that many concepts that have been developed in the context of NMR spectroscopy and are rooted in Redfield theory are also relevant in the context of ESR spectroscopy. For example, we know that in NMR asymmetric doublets arise from cross-correlations between the chemical shift anisotropy (CSA) and dipolar interactions. In the case of ESR spectroscopy, the anisotropic portion of the Zeeman interaction is equivalent to the CSA interaction, while the anisotropic portion of the hyperfine interaction is analogous to the dipolar interaction. Hence, it can be predicted that the asymmetric ESR doublets, such as seen in Fig. 4, can be attributed to the cross-correlation between the ${{\bf{g}}}_{aniso}$ and ${{\bf{A}}}_{aniso}$ terms in the spin-Hamiltonian of the system, Eq. (1).

This prediction is rather easy to verify. Indeed, when calculating Redfield matrix one can “switch off” the corresponding cross-correlated contributions (i.e. forcibly set these terms to zero). This manipulation renders the ESR doublets symmetric, see Fig. S14. Hence, the observed asymmetry can be unequivocally attributed to the discussed cross-correlation effect.

It should be noted that cross-correlations in ESR have a long history. In fact, this phenomenon had been first discovered and rationalized in the context of ESR spectroscopy^97–99^, before it was described in NMR^100^. Yet in this paper we demonstrate this effect for the first time (i) in the context of *protein* ESR spectroscopy and, more specifically, (ii) for protein samples labeled by ^15^N-enriched MTSSL, (iii) in relation to the MD-based calculations of the ESR lineshapes and (iv) using modern Liouville-space formulation of the Redfield theory. The combination of the rigorous direct propagation treatment and the Redfield scheme makes it possible to establish the range of validity of the Redfield theory. This range appears to be much broader than previously believed, covering not only small paramagnetic molecules, but also many spin-labeled protein samples of practical interest. The analogy with the renowned TROSY effect raises an intriguing possibility that the concept of TROSY experiment may someday be adapted for use in pulsed ESR spectroscopy.

## Conclusion

Nitroxyl spin labels proved to be useful reporters of local protein environment and dynamics. In broad, qualitative terms, the relationship between the ESR spectra and the underlying structural/dynamic factors is well understood. However, at the more detailed level the picture remains fuzzy. The ESR lineshapes are not necessarily rich in details and, therefore, it is difficult to uniquely interpret them in terms of complex dynamic behavior of R1-tagged protein molecules. In other words, the problem is intrinsically underdetermined. In this situation, attempts at quantitative interpretation of ESR data typically involve various approximations and assumptions.

The progress in the field of MD simulations and development of new methods to calculate ESR spectra based on the MD data should change this situation. In our study, we have recorded MD trajectories for seven different spin-labeled variants of the protein GB1 (net duration 180 μs) and two variants of free spin label (additional 10 μs). This is two orders of magnitude longer than previously employed in the context of ESR simulations. The MD-based calculations predict ESR spectra in good agreement with our experimental measurements.

We have confirmed the existing notion that the shape of the spectrum is primarily dictated by steric confinement of the spin label (R1 tag) in the protein matrix. Specifically, the lineshape is largely dependent on the degree of R1 exposure to solvent. Accordingly, we found that the ESR lineshape descriptors correlate well with solvent accessible surface area of the R1 side chain.

We have made an effort to design GB1 constructs that would accommodate the R1 tag in their hydrophobic core. However, our experimental ESR and NMR data suggest that these samples suffer from lowered stability and partial unfolding effects. This led us to focus on the constructs where R1 tag is projected into solvent (such constructs are typically used in practical applications). For those samples, we have found that preferred $({\chi }_{1},{\chi }_{2})$ rotameric states of the R1 side chain are dictated mainly by the backbone topology (α-helix vs. β-sheet) and not by unconventional weak interactions such as S^δ^···C^α^-H^α^. The rotameric jumps involving ${\chi }_{1}$ and ${\chi }_{2}$ are, in fact, sufficiently fast to directly influence the ESR lineshape. However, the effect of ${\chi }_{i}$ jumps on the motion of the paramagnetic center is significantly less than may be expected. This is because the jumps involving several torsional angles ${\chi }_{i}$ tend to occur in (anti)correlated manner, causing only a relatively small movement of the proxyl ring. In this sense, the R1 side chain plays a role of a mobile linker that connects the heavy proxyl ring to the body of the protein.

For stable well-folded proteins with R1 attachment sites in α-helical or β-sheet regions, ESR spectra contain little direct information about backbone dynamics. Indeed, the amplitudes of backbone dynamics at such sites are (uniformly) small, with backbone order parameters confined to a narrow range *S*^2^ = 0.80–0.90. Furthermore, backbone motions are not directly transmitted to the proxyl ring because their effect is damped by the intervening flexible R1 side chain. While SDSL ESR can successfully discriminate between protein order and disorder^101–104^, it is not well suited to quantify small variations in *S*^2^ within the fairly rigid protein scaffold.

The sensitivity to backbone dynamics can be conceivably improved by using more rigid bifunctional spin labels^105^, but these labels retain certain degree of conformational mobility and, besides, are more likely to perturb the system’s native dynamics. Similar to what has been described above, such more advanced spin-labeling strategies also stand to benefit from MD-assisted studies.

Our simulations have also shown that R1 tag can sample multiple different sites (environments) on the protein surface, remaining in these distinctive states for a long time (microseconds). In principle, this should give rise to multicomponent ESR spectra. However, visually they cannot be readily identified as such (only dramatic cases, such as samples containing a mixture of folded and unfolded species, produce an easily identifiable multicomponent lineshapes). Proper investigation of such systems requires special efforts, e.g. variable-temperature measurements.

We have also taken advantage of a unique opportunity to test the validity of Redfield theory for spin-labeled protein samples. To this end, we have compared the spectra simulated by means of the rigorous direct propagation scheme with those obtained by Redfield-theory calculations (based on the same MD trajectories). The results suggest that Redfield theory should work well for many protein samples, especially for those samples where R1 tag is attached to the surface of the protein.

In turn, this means that many spectroscopic concepts that are rooted in Redfield theory and have been actively developed in the field of NMR spectroscopy can be successfully adapted for use in protein ESR. To illustrate this point, we have manufactured several samples of GB1 labeled with ^15^N-enriched MTSSL. The absorption-mode ESR spectra of these samples have the appearance of asymmetric doublets, similar to NMR doublets best known in the context of the TROSY experiment. We have confirmed that this spectral pattern arises from the Redfield-type cross-correlation that is equivalent to the CSA-dipolar cross-correlation in NMR.

Finally, it is worth noting that MD-based studies offer some remarkable possibilities to elucidate the origin of certain spectral features. For instance, we were able to “switch off” the cross-correlation effects and verify that this leads to a disappearance of the asymmetry in the simulated ESR spectrum. Along the same lines, using special MD processing algorithm we were able to increase/decrease the rate of the protein tumbling and thus probe its influence on the shape of the spectrum.

The MD-based, experimentally validated ESR studies, such as presented in this paper, have many exciting future applications. For example, this approach can be extended to multifrequency ESR studies (X-band, W-band, etc.), allowing for much better grasp on the details of motion^106^. Such future study can be combined with MD-assisted analyses of NMR paramagnetic relaxation enhancements, which are known to be sensitive to conformational dynamics of the nitroxide label^107,108^. It should also be interesting to address the use of viscogens (such as glycerol) in ESR samples. Originally, it was suggested that viscogens suppress the protein overall tumbling without affecting the conformational mobility of the R1 tag^2^. An MD simulation of a glycerol-containing sample can put this assumption to a test, which is especially relevant for those systems where the tag is extended into solvent and thus comes in direct contact with glycerol. Separately, it should be pointed out that MD-based approach can be used to model ESR spectra of crystalline proteins, amyloid fibrils, precipitated proteins, etc. The power of modern GPU-equipped computers allows one to model even a large unit crystal cell or a big cluster of protein molecules, representing the precipitated state. Finally, the MD-based method employing direct propagation scheme should be highly useful for interpretation of DEER experiments. This area has been the forefront of the biomolecular ESR spectroscopy and the effort has been underway to put the analyses of DEER data on a more solid quantitative basis^27,28,109,110^. These efforts should greatly benefit from the rigorous simulation strategy such as demonstrated in our work.

## Materials and Methods

### Sample preparation

Seven single-cysteine mutants of GB1 domain were expressed in Rosetta (DE3) cells transformed with pET-28 or pET-24a(+) plasmids and grown in LB media (or minimal M9 media in the case of ^15^N-enriched sample GB1 F30C). Protein expression and purification protocol was the same as reported previously^56^, with the following minor modifications. The harvested cells were frozen and then lysed in the lysis buffer (20 mM Tris-HCl, 5 mM NaCl, 1 mM benzamidine, 5 mM DTT, 0.1 mM PMSF at pH 8.5) using SPEX SamplePrep 6870 Freezer/Mill, followed by three cycles of sonication on ice. Size-exclusion chromatography was performed using GE Sephacryl S-200 HR column pre-equilibrated with 30 mM ammonium bicarbonate buffer containing 1 mM DTT. The purified protein material was flash-frozen in liquid nitrogen, lyophilized and stored at −80 °C until needed.

MTSSL labeling was conducted as described previously^56^ using GB1 solution with concentration 40–70 μM and labeling reagent excess 10:1 (regular MTSSL) or 5:1 (^15^N-enriched MTSSL). In the case of F30R1, we have tested two alternative protocols: (i) labeling was conducted in solution with 8 M urea to ensure good access to the labeling site or (ii) labeled protein was purified by ion-exchange chromatography with the intent to get rid of misfolded species, followed by the (standard) application of desalting column.

CW ESR spectrum of MTSSL^15N^ (Toronto Research Chemicals, catalogue number O875002) featured a broad signal from a certain unidentified contamination. In order to obtain a contaminant-free sample, we labeled GB1 T44C with MTSSL^15N^, removed the excess labeling reagent as well as low molecular weight impurities by ultrafiltration, and reduced the resulting T44R1^15N^ material (2 h incubation with 5 mM DTT) to detach the spin label in a form of ${{\rm{MTSSL}}}_{\Delta }^{{\rm{15N}}}$. In this manner, we have obtained the sample containing 76 μM of reduced T44C GB1 and the same amount of free ${{\rm{MTSSL}}}_{\Delta }^{{\rm{15N}}}$, i.e. ^15^N-(1-oxyl-2,2,5,5-tetramethylpyrroline-3-methyl)-methanethiol. This sample, without further separation steps, was used to record the ESR spectrum of ${{\rm{MTSSL}}}_{\Delta }^{{\rm{15N}}}$, which was found to be artefact-free, see Fig. 4a.

Protein samples for ESR measurements were prepared in a buffer solution with 50 mM sodium phosphate, 150 mM NaCl at pH 6.5. Before transferring the sample to the ESR tube, the solution was centrifuged for 10 minutes at 20,000 g to remove air bubbles. The concentration of the protein in the samples ranged from 45 to 100 μM, the concentration of free MTSSL was 50 μM. An additional sample with concentration of free MTSSL 2 mM was prepared to assess the effect of intermolecular hyperfine interaction (the effect was found to be negligible). Samples were placed in quartz capillary tubes with inner diameter 1 mm and wall thickness 0.5 mm; the volume of each sample was ca. 30 μL. To prepare deoxygenated sample of free MTSSL, the ESR tube containing the sample was connected to Schlenk line and subjected to four cycles of freeze-pump-thaw procedure under 10^−5^ bar vacuum. After that, the tube was immediately sealed and used to acquire the ESR spectrum. The efficiency of this method was tested in a separate NMR experiment where we measured spin-lattice relaxation of residual HDO signal in the D_2_O sample (*T*_1_ = 14.5 s in air-equilibrated sample vs. 44.7 s in degassed sample).

### ESR measurements and spectral processing

CW ESR spectra were recorded using the X-band (9.45 GHz) Bruker Elexsys E580 spectrometer with the following experimental settings: sweep width 12 mT; sweep time 120 s; microwave power 0.7518 mW; modulation frequency 100 kHz; modulation amplitude 0.05 mT; number of points 8192; number of scans 25; temperature 293 K (stabilized by N_2_ gas flow to within 1 K). For samples prepared with MTSSL^15N^ reagent, the following two parameters have been changed: sweep width 10 mT; sweep time 300 s.

All spectral processing was performed using python scripts written in-house. Linear baseline correction has been applied to the first-derivative spectra; the correction was calculated using the spectral intervals [−6.0 mT, −3.5 mT] and [3.5 mT, 6.0 mT] (in the case of samples containing ^15^N-proxyl, [−5.0 mT, −3.5 mT] and [3.5 mT, 5.0 mT]). The absorption-mode spectra are more sensitive to small phase imperfections than first-derivative spectra. To address this issue, the experimental spectra in Fig. 4 were additionally phase-corrected by means of the standard algorithm involving Hilbert transform^111^. The applied phase corrections were 0°, −4° and −5° for free ${{\rm{MTSSL}}}_{\Delta }^{{\rm{15N}}}$, N8R1^15N^ and T44R1^15N^, respectively.

To obtain a handle on additional sources of ESR line broadening, we have analyzed the spectra of air-equilibrated and degassed free MTSSL. The lines in these two spectra were fitted using the first derivative of the Voigt function and assuming that there are three sources of broadening: (L) Lorentzian contribution due to spin relaxation via anisotropic g-tensor and hyperfine coupling; (L_O2_) Lorentzian contribution due to paramagnetic oxygen; (G) Gaussian contribution due to static magnetic field inhomogeneity as well as unresolved hyperfine couplings to remote ^1^H spins and ^13^C spins (at natural abundance)^112^. Using these conventions, the three lines in the spectrum of free MTSSL are characterized by the following parameters: (L_(1)_ + L_O2_,G), (L_(0)_ + L_O2_,G) and (L_(−1)_ + L_O2_,G). In the case of degassed sample, the set of parameters is reduced to (L_(1)_,G), (L_(0)_,G) and (L_(−1)_,G). Collective fitting of both spectra yields full width at half height 126 μT for the Gaussian contour G and 13.5 μT for the Lorentzian contour L_O2_ (corresponding to ${T}_{2}^{{\rm{O2}}}=0.83\,{\rm{\mu }}{\rm{s}}$ for oxygen-induced relaxation of the electron spin). The fitting procedure was implemented such that each peak was fitted over the interval between (i) the leftmost point corresponding to 80% of the maximal peak intensity and (ii) the rightmost point corresponding to 80% of the minimal peak intensity. The results are illustrated in Fig. S2.

To account for contribution of these extra broadening mechanisms to GB1 lineshapes, we begin with $FID(\tau )$, multiply it by $\exp (-\,{\tau /T}_{2}^{{\rm{O2}}})$, calculate the spectrum according to Eq. (5) and then convolute it with the Gaussian contour G described above. Note that our procedure to estimate O_2_-induced broadening in the GB1 spectra involves two approximations. First, it is appropriate only for those samples where R1 tag is fully exposed to solvent. For samples with the buried tag, such as Y3R1 and F30R1, the contribution from O_2_ should be scaled down. Second, it implies that the O_2_ mechanism is dominated by fast oxygen diffusion and local R1 dynamics. Considering that molecular diffusion of spin-labeled GB1 is slower than that of the free spin label, the O_2_ contribution should be revised slightly upward. However, given that (*i*) the contribution from paramagnetic oxygen is very small relative to other terms, (*ii*) we do not pursue a quantitative comparison between the predicted and experimental spectra for Y3R1 or F30R1, and (*iii*) the two described sources of error should to some degree compensate each other, we did not attempt to correct for these (extremely subtle) effects.

To obtain Δ_(0)_ and ${h}_{(0)}/{h}_{(-1)}$ parameters of the first-derivative spectra, we use a similar fitting procedure whereupon each spectral line is fitted with the Voigt contour. The frequency separation between the maximum and the minimum of the best-fit function is taken to be the width of the line Δ_(*i*)_. The difference between the maximal and the minimal intensity of the best-fit function is taken to be the amplitude of the line ${h}_{(i)}$.

### MD simulations and processing of MD trajectories

Initial coordinates for MD simulations were prepared using the PDB structures 5bmg (T44R1), 5bmh (E15R1) or 1pgb (all other constructs). To introduce cysteine residue into desired position, the latter structure was subjected to *in silico* mutagenesis. Likewise, the R1 tag was attached *in silico*, with ${\chi }_{i}$ angles adjusted such as to avoid steric clashes. Structures were protonated using the program PROPKA^113^, assuming the same pH as in our experimental study, pH 6.5, and then solvated with >10 Å thick TIP3P^43^ water shell. The number of water molecules in the truncated octahedral cell varied from 3,196 to 4,363 (the higher numbers correspond to outward-projected R1 tag). In each case, the cell was neutralized with Na^+^ ions.

The simulations were conducted using Amber v16 and v18 program under Amber ff14SB force field^42^. The R1 geometry and force field parameters were as reported previously^44,45^. Following the solvation step, the system was energy-minimized, heated to 293 K and then equilibrated in a standard fashion. The production run was conducted with an NPT ensemble. The numerical integration of the equations of motions was performed using the leapfrog algorithm with a time step of 2 fs. Bonds involving hydrogens have been restrained by means of the SHAKE algorithm^114^. The non-bonded interactions were calculated with a cutoff of 10.5 Å^115^. The particle mesh Ewald summation scheme has been employed to treat long-range electrostatic interactions with the default parameters for grid spacing and spline interpolation. Pressure coupling was achieved with a Berendsen barostat^116^ with a pressure relaxation time *τ*_*p*_ = 2 ps. The Langevin thermostat^117^ was used with the collision frequency *γ* = 2 ps^−1^. The protein coordinates were stored every 1 ps. The simulations were conducted using in-house GPU workstations under CUDA MPS. The highest production rate, 184 ns/day, was obtained using GTX 1080 Ti card. The stability of the simulations (as well as occurrence of certain transient conformational states) is illustrated by the *rmsd* traces, Fig. S7.

To record the trajectories of free MTSSL and MTSSL_Δ_, the necessary force field parameters were obtained in a conventional fashion. The known geometry and parameters for R1 were used as a starting point^44,45^. Following geometry optimization, the electrostatic potentials of MTSSL and MTSSL_Δ_ were calculated in Gaussian 16^118^ by means of the Hartree-Fock method using 6–31 G(d) basis set^119^. The RESP algorithm has been employed to obtain point charges for the terminal (methanethiosulfonate or thiol) groups^120^. Other parameters were taken from gaff2 force field^121^ as needed. The resulting library and frcmod files can be found at https://github.com/bionmr-spbu-projects/2019-GB1-ESR. The MD simulations for free MTSSL and MTSSL_Δ_ have been set up same as described above, with 5 μs trajectories recorded for both molecules.

The MD data have been processed using python library written in-house, pyxmolpp2, also available at https://github.com/bionmr-spbu/pyxmolpp2. Solvent-accessible surface area of R1 side chain has been computed on per-frame basis for all side-chain atoms (beginning with C^β^) using Lee and Richards algorithm^122^ implemented in pyxmolpp2 with default probe radius 1.4 Å. The occurrence of hydrogen bonds and secondary structure in the trajectories has been quantified using the programs *hbplus*^123^ and STRIDE^124^, respectively. Other details are described in the text and in the SI.

## Supplementary information

Supplementary information.

## Data Availability

All data are available from the authors upon request.

## References

[1] Altenbach C, Flitsch SL, Khorana HG, Hubbell WL (1989). Structural studies of transmembrane proteins. 2. Spin labeling of bacteriorhodopsin mutants at unique cysteines. Biochemistry.

[2] Mchaourab HS, Lietzow MA, Hideg K, Hubbell WL (1996). Motion of spin-labeled side chains in T4 lysozyme. Correlation with protein structure and dynamics. Biochemistry.

[3] *Spin labeling: theory and applications*. (ed. Berliner, L. J.) (Academic Press, 1976).

[4] Kim JM (2004). Structural origins of constitutive activation in rhodopsin: role of the K296/E113 salt bridge. Proc Natl Acad Sci USA.

[5] Cordero-Morales JF (2006). Molecular determinants of gating at the potassium-channel selectivity filter. Nat Struct Mol Biol.

[6] Dong JH, Yang GY, Mchaourab HS (2005). Structural basis of energy transduction in the transport cycle of MsbA. Science.

[7] Stone KM (2013). Structural insight into proteorhodopsin oligomers. Biophys J.

[8] Yu, L. *et al*. CW-EPR studies revealed different motional properties and oligomeric states of the integrin β_1a_ transmembrane domain in detergent micelles or liposomes. *Sci Rep* **5**, 10.1038/srep07848 (2015). 10.1038/srep07848 PMC4297981 25597475

[9] Zhang YH, Shin YK (2006). Transmembrane organization of yeast syntaxin-analogue Sso1p. Biochemistry.

[10] Galazzo, L. *et al*. Identifying conformational changes with site-directed spin labeling reveals that the GTPase domain of HydF is a molecular switch. *Sci Rep* **7**, 10.1038/s41598-017-01886-y (2017). 10.1038/s41598-017-01886-y PMC5431965 28490758

[11] Lawless MJ, Pettersson JR, Rule GS, Lanni F, Saxena S (2018). ESR resolves the C terminus structure of the ligand-free human glutathione S-transferase A1-1. Biophys J.

[12] Mchaourab HS, Oh KJ, Fang CJ, Hubbell WL (1997). Conformation of T4 lysozyme in solution. Hinge-bending motion and the substrate-induced conformational transition studied by site-directed spin labeling. Biochemistry.

[13] Davydov DR, Yang ZY, Davydova N, Halpert JR, Hubbell WL (2016). Conformational mobility in cytochrome P450 3A4 explored by pressure-perturbation EPR spectroscopy. Biophys J.

[14] Belle V (2007). Probing the opening of the pancreatic lipase lid using site-directed spin labeling and EPR spectroscopy. Biochemistry.

[15] Agafonov RV, Nesmelov YE, Titus MA, Thomas DD (2008). Muscle and nonmuscle myosins probed by a spin label at equivalent sites in the force-generating domain. Proc Natl Acad Sci USA.

[16] Sugata K, Nakamura M, Ueki S, Fajer PG, Arata T (2004). ESR reveals the mobility of the neck linker in dimeric kinesin. Biochem Biophys Res Comm.

[17] Polnaszek CF, Freed JH (1975). Electron spin resonance studies of anisotropic ordering, spin relaxation, and slow tumbling in liquid crystalline solvents. J Phys Chem.

[18] Liang ZC, Freed JH (1999). An assessment of the applicability of multifrequency ESR to study the complex dynamics of biomolecules. J Phys Chem B.

[19] Hubbell WL, Mchaourab HS, Altenbach C, Lietzow MA (1996). Watching proteins move using site-directed spin labeling. Structure.

[20] Lietzow MA, Hubbell WL (2004). Motion of spin label side chains in cellular retinol-binding protein: correlation with structure and nearest-neighbor interactions in an antiparallel beta-sheet. Biochemistry.

[21] Guo ZF, Cascio D, Hideg K, Kalai T, Hubbell WL (2007). Structural determinants of nitroxide motion in spin-labeled proteins: tertiary contact and solvent-inaccessible sites in helix G of T4 lysozyme. Protein Sci.

[22] Mchaourab HS, Kalai T, Hideg K, Hubbell WL (1999). Motion of spin-labeled side chains in T4 lysozyme: effect of side chain structure. Biochemistry.

[23] Langen R, Oh KJ, Cascio D, Hubbell WL (2000). Crystal structures of spin labeled T4 lysozyme mutants: implications for the interpretation of EPR spectra in terms of structure. Biochemistry.

[24] Guo ZF, Cascio D, Hideg K, Hubbell WL (2008). Structural determinants of nitroxide motion in spin-labeled proteins: solvent-exposed sites in helix B of T4 lysozyme. Protein Sci.

[25] Stoica I (2005). Force field impact and spin-probe modeling in molecular dynamics simulations of spin-labeled T4 lysozyme. J Mol Model.

[26] Ding F, Layten M, Simmerling C (2008). Solution structure of HIV-1 protease flaps probed by comparison of molecular dynamics simulation ensembles and EPR experiments. J Am Chem Soc.

[27] Polyhach Y, Bordignon E, Jeschke G (2011). Rotamer libraries of spin labelled cysteines for protein studies. Phys Chem Chem Phys.

[28] Jeschke, G. DEER distance measurements on proteins. In *Annual Review of Physical Chemistry* Vol. 63 (eds Johnson, M. A. & Martinez, T. J.) 419–446 (2012). 10.1146/annurev-physchem-032511-143716 22404592

[29] Steinhoff HJ, Hubbell WL (1996). Calculation of electron paramagnetic resonance spectra from Brownian dynamics trajectories: application to nitroxide side chains in proteins. Biophys J.

[30] Budil DE, Sale KL, Khairy KA, Fajer PG (2006). Calculating slow-motional electron paramagnetic resonance spectra from molecular dynamics using a diffusion operator approach. J Phys Chem A.

[31] Freed, J. H. Theory of slow tumbling ESR spectra for nitroxides. in *Spin labelling: theory and applications* (ed Berliner, L. J.) Ch. 3, 53–132 (Academic Press, 1976).

[32] Sezer D, Freed JH, Roux B (2008). Using Markov models to simulate electron spin resonance spectra from molecular dynamics trajectories. J Phys Chem B.

[33] Sezer D, Freed JH, Roux B (2009). Multifrequency electron spin resonance spectra of a spin-labeled protein calculated from Molecular Dynamics simulations. J Am Chem Soc.

[34] Tyrrell, S. & Oganesyan, V. S. Simulation of electron paramagnetic resonance spectra of spin-labeled molecules from replica-exchange molecular dynamics. *Phys Rev E* **88**, 10.1103/PhysRevE.88.042701 (2013). 10.1103/PhysRevE.88.042701 24229207

[35] Håkansson P, Westlund PO, Lindahl E, Edholm O (2001). A direct simulation of EPR slow-motion spectra of spin labelled phospholipids in liquid crystalline bilayers based on a molecular dynamics simulation of the lipid dynamics. Phys Chem Chem Phys.

[36] DeSensi SC, Rangel DP, Beth AH, Lybrand TP, Hustedt EJ (2008). Simulation of nitroxide electron paramagnetic resonance spectra from Brownian trajectories and molecular dynamics simulations. Biophys J.

[37] Oganesyan VS, Chami F, White GF, Thomson AJ (2017). A combined EPR and MD simulation study of a nitroxyl spin label with restricted internal mobility sensitive to protein dynamics. J Magn Reson.

[38] Alexander P, Fahnestock S, Lee T, Orban J, Bryan P (1992). Thermodynamic analysis of the folding of the streptococcal protein G IgG-binding domains B1 and B2: why small proteins tend to have high denaturation temperatures. Biochemistry.

[39] Zhou DH (2007). Solid-state protein-structure determination with proton-detected triple-resonance 3D magic-angle-spinning NMR spectroscopy. Angew Chem, Int Ed.

[40] Gronenborn AM (1991). A novel, highly stable fold of the immunoglobulin binding domain of streptococcal protein G. Science.

[41] Case, D. A. *et al*. *AMBER 16*. (University of California, 2016).

[42] Maier JA (2015). ff14SB: improving the accuracy of protein side chain and backbone parameters from ff99SB. J Chem Theory Comput.

[43] Jorgensen WL, Chandrasekhar J, Madura JD, Impey RW, Klein ML (1983). Comparison of simple potential functions for simulating liquid water. J Chem Phys.

[44] Sezer D, Freed JH, Roux B (2008). Parametrization, molecular dynamics simulation, and calculation of electron spin resonance spectra of a nitroxide spin label on a polyalanine α-helix. J Phys Chem B.

[45] Xue Y, Skrynnikov NR (2011). Motion of a disordered polypeptide chain as studied by paramagnetic relaxation enhancements, ^15^N relaxation, and Molecular Dynamics simulations: how fast is segmental diffusion in denatured ubiquitin?. J Am Chem Soc.

[46] Hyde JS, Pasenkiewicz-Gierula M, Lesmanowicz A, Antholine WE (1990). Pseudo field modulation in EPR spectroscopy. Appl Magn Reson.

[47] Hyde JS, Jesmanowicz A, Ratke JJ, Antholine WE (1992). Pseudomodulation: a computer-based strategy for resolution enhancement. J Magn Reson.

[48] Altenbach C, Lopez CJ, Hideg K, Hubbell WL (2015). Exploring structure, dynamics, and topology of nitroxide spin-labeled proteins using continuous-wave Electron Paramagnetic Resonance spectroscopy. Methods Enzymol.

[49] Pirman NL, Milshteyn E, Galiano L, Hewlett JC, Fanucci GE (2011). Characterization of the disordered-to-α-helical transition of IA_3_ by SDSL-EPR spectroscopy. Protein Sci.

[50] Seewald MJ (2000). The role of backbone conformational heat capacity in protein stability: temperature-dependent dynamics of the B1 domain of Streptococcal protein G. Protein Sci.

[51] Sheppard D, Li DW, Bruschweiler R, Tugarinov V (2009). Deuterium spin probes of backbone order in proteins: ^2^H NMR relaxation study of deuterated carbon α sites. J Am Chem Soc.

[52] de la Torre JG, Huertas ML, Carrasco B (2000). HYDRONMR: Prediction of NMR relaxation of globular proteins from atomic-level structures and hydrodynamic calculations. J Magn Reson Ser B.

[53] Davey JA, Damry AM, Goto NK, Chica RA (2017). Rational design of proteins that exchange on functional timescales. Nat Chem Biol.

[54] Jee J, Byeon I-JL, Louis JM, Gronenborn AM (2008). The point mutation A34F causes dimerization of GB1. Proteins.

[55] Louis JM, Byeon IJL, Baxal U, Gronenborn AM (2005). The GB1 amyloid fibril: recruitment of the peripheral beta-strands of the domain swapped dimer into the polymeric interface. J Mol Biol.

[56] Cunningham TF (2012). High-resolution structure of a protein spin-label in a solvent-exposed β-sheet and comparison with DEER spectroscopy. Biochemistry.

[57] Frank MK, Dyda F, Dobrodumov A, Gronenborn AM (2002). Core mutations switch monomeric protein GB1 into an intertwined tetramer. Nat Struct Biol.

[58] Kuszewski J, Clore GM, Gronenborn AM (1994). Fast folding of a prototypic polypeptide: the immunoglobulin binding domain of streptococcal protein G. Protein Sci.

[59] Lindorff-Larsen K, Piana S, Dror RO, Shaw DE (2011). How fast-folding proteins fold. Science.

[60] Piana S, Lindorff-Larsen K, Shaw DE (2013). Atomic-level description of ubiquitin folding. Proc Natl Acad Sci USA.

[61] Wright JD, Lim C (2001). A fast method for predicting amino acid mutations that lead to unfolding. Protein Eng Des Sel.

[62] Bershtein S, Mu WM, Shakhnovich EI (2012). Soluble oligomerization provides a beneficial fitness effect on destabilizing mutations. Proc Natl Acad Sci USA.

[63] Zou P, Mchaourab HS (2009). Alternating access of the putative substrate-binding chamber in the ABC transporter MsbA. J Mol Biol.

[64] Bridges MD, Hideg K, Hubbell WL (2010). Resolving conformational and rotameric exchange in spin-labeled proteins using saturation recovery EPR. Appl Magn Reson.

[65] Nabuurs SM, de Kort BJ, Westphal AH, van Mierlo CPM (2010). Non-native hydrophobic interactions detected in unfolded apoflavodoxin by paramagnetic relaxation enhancement. Eur Biophys J.

[66] Fanucci GE, Cafiso DS (2006). Recent advances and applications of site-directed spin labeling. Curr Opin Struct Biol.

[67] Lopez CJ, Oga S, Hubbell WL (2012). Mapping molecular flexibility of proteins with site-directed spin labeling: a case study of myoglobin. Biochemistry.

[68] Columbus L, Kalai T, Jeko J, Hideg K, Hubbell WL (2001). Molecular motion of spin labeled side chains in α-helices: analysis by variation of side chain structure. Biochemistry.

[69] Bouvignies G, Markwick P, Bruschweiler R, Blackledge M (2006). Simultaneous determination of protein backbone structure and dynamics from residual dipolar couplings. J Am Chem Soc.

[70] Bouvignies G (2005). Identification of slow correlated motions in proteins using residual dipolar and hydrogen-bond scalar couplings. Proc Natl Acad Sci USA.

[71] Clore GM, Schwieters CD (2004). Amplitudes of protein backbone dynamics and correlated motions in a small alpha/beta protein: correspondence of dipolar coupling and heteronuclear relaxation measurements. Biochemistry.

[72] Gallagher T, Alexander P, Bryan P, Gilliland GL (1994). Two crystal structures of the B1 immunoglobulin-binding domain of streptococcal protein-G and comparison with NMR. Biochemistry.

[73] Fleissner MR, Cascio D, Hubbell WL (2009). Structural origin of weakly ordered nitroxide motion in spin-labeled proteins. Protein Sci.

[74] Kroncke BM, Horanyi PS, Columbus L (2010). Structural origins of nitroxide side chain dynamics on membrane protein α-helical sites. Biochemistry.

[75] Pliotas C (2015). The role of lipids in mechanosensation. Nat Struct Mol Biol.

[76] Warshaviak DT, Serbulea L, Houk KN, Hubbell WL (2011). Conformational analysis of a nitroxide side chain in an α-helix with density functional theory. J Phys Chem B.

[77] Freed DM, Khan AK, Horanyi PS, Cafiso DS (2011). Molecular origin of electron paramagnetic resonance line shapes on β-barrel membrane proteins: the local solvation environment modulates spin-label configuration. Biochemistry.

[78] Florin, N., Schiemann, O. & Hagelueken, G. High-resolution crystal structure of spin labelled (T21R1) azurin from Pseudomonas aeruginosa: a challenging structural benchmark for in silico spin labelling algorithms. *BMC Struct Biol* **14**, 10.1186/1472-6807-14-16 (2014). 10.1186/1472-6807-14-16 PMC4055355 24884565

[79] Cunningham TF, Pornsuwan S, Horne WS, Saxena S (2016). Rotameric preferences of a protein spin label at edge-strand β-sheet sites. Protein Sci.

[80] Abdullin D, Hagelueken G, Schiemann O (2016). Determination of nitroxide spin label conformations via PELDOR and X-ray crystallography. Phys Chem Chem Phys.

[81] Carrington B, Myers WK, Horanyi P, Calmiano M, Lawson ADG (2017). Natural conformational sampling of human TNFα visualized by double electron-electron resonance. Biophys J.

[82] Mo YR (2011). Rotational barriers in alkanes. Wiley Interdiscip Rev Comput Mol Sci.

[83] Cousin SF (2018). Time-resolved protein side-chain motions unraveled by high-resolution relaxometry and Molecular Dynamics simulations. J Am Chem Soc.

[84] Fraser RR, Boussard G, Saunders JK, Lambert JB, Mixan CE (1971). Barriers to rotation about the sulfur-sulfur bond in acyclic disulfides. J Am Chem Soc.

[85] Trbovic N (2009). Protein side-chain dynamics and residual conformational entropy. J Am Chem Soc.

[86] Kamberaj H, van der Vaart A (2009). Extracting the causality of correlated motions from Molecular Dynamics simulations. Biophys J.

[87] Tiberti M, Invernizzi G, Papaleo E (2015). (Dis)similarity index to compare correlated motions in molecular simulations. J Chem Theory Comput.

[88] Anderson JS, LeMaster DM (2012). Rotational velocity rescaling of molecular dynamics trajectories for direct prediction of protein NMR relaxation. Biophys Chem.

[89] Showalter SA, Bruschweiler R (2007). Validation of molecular dynamics simulations of biomolecules using NMR spin relaxation as benchmarks: application to the AMBER99SB force field. J Chem Theory Comput.

[90] Goldman M (1984). Interference effects in the relaxation of a pair of unlike spin-1/2 nuclei. J Magn Reson.

[91] Tjandra N, Szabo A, Bax A (1996). Protein backbone dynamics and ^15^N chemical shift anisotropy from quantitative measurement of relaxation interference effects. J Am Chem Soc.

[92] Pervushin K, Riek R, Wider G, Wüthrich K (1997). Attenuated T_2_ relaxation by mutual cancellation of dipole-dipole coupling and chemical shift anisotropy indicates an avenue to NMR structures of very large biological macromolecules in solution. Proc Natl Acad Sci USA.

[93] Redfield AG (1957). On the theory of relaxation processes. IBM J Res Dev.

[94] Ernst, R. R., Bodenhausen, G. & Wokaun, A. *Principles of nuclear magnetic resonance in one and two dimensions*. (Oxford University Press, 1987).

[95] Brüschweiler R (1992). Influence of rapid intramolecular motion on NMR cross-relaxation rates. A Molecular Dynamics study of antamanide in solution. J Am Chem Soc.

[96] Kowalewski, J. & Maler, L. *Nuclear spin relaxation in liquids: theory, experiments, and applications (second edition)*. (CRC Press, 2017).

[97] McConnell HM (1956). Effect of anisotropic hyperfine interactions on paramagnetic relaxation in liquids. J Chem Phys.

[98] Rogers RN, Pake GE (1960). Paramagnetic relaxation in solutions of VO^++^. J Chem Phys.

[99] Freed JH, Fraenkel GK (1963). Theory of linewidths in Electron Spin Resonance spectra. J Chem Phys.

[100] Shimizu H (1964). Theory of the dependence of nuclear magnetic relaxation on the absolute sign of spin-spin coupling constant. J Chem Phys.

[101] Lo RH, Kroncke BM, Solomon TL, Columbus L (2014). Mapping membrane protein backbone dynamics: a comparison of site-directed spin labeling with NMR ^15^N relaxation measurements. Biophys J.

[102] Columbus L, Hubbell WL (2004). Mapping backbone dynamics in solution with site-directed spin labeling: GCN4-58 bZip free and bound to DNA. Biochemistry.

[103] Der-Sarkissian A, Jao CC, Chen J, Langen R (2003). Structural organization of α-synuclein fibrils studied by site-directed spin labeling. J Biol Chem.

[104] Hanson SM (2006). Differential interaction of spin-labeled arrestin with inactive and active phosphorhodopsin. Proc Natl Acad Sci USA.

[105] Fleissner MR (2011). Structure and dynamics of a conformationally constrained nitroxide side chain and applications in EPR spectroscopy. Proc Natl Acad Sci USA.

[106] Nesmelov YE, Thomas DD (2010). Protein structural dynamics revealed by site-directed spin labeling and multifrequency EPR. Biophys Rev.

[107] Iwahara J, Schwieters CD, Clore GM (2004). Ensemble approach for NMR structure refinement against ^1^H paramagnetic relaxation enhancement data arising from a flexible paramagnetic group attached to a macromolecule. J Am Chem Soc.

[108] Xue Y (2009). Paramagnetic relaxation enhancements in unfolded proteins: Theory and application to drkN SH3 domain. Protein Sci.

[109] Islam SM, Roux B (2015). Simulating the distance distribution between spin-labels attached to proteins. J Phys Chem B.

[110] Worswick SG, Spencer JA, Jeschke G, Kuprov I (2018). Deep neural network processing of DEER data. Sci Adv.

[111] Ernst RR (1969). Numerical Hilbert transform and automatic phase correction in magnetic resonance spectroscopy. J Magn Reson.

[112] Bales BL, Peric M, Lamy-Freund MT (1998). Contributions to the Gaussian line broadening of the proxyl spin probe EPR spectrum due to magnetic-field modulation and unresolved proton hyperfine structure. J Magn Reson.

[113] Bas DC, Rogers DM, Jensen JH (2008). Very fast prediction and rationalization of pK_a_ values for protein-ligand complexes. Proteins.

[114] Ryckaert JP, Ciccotti G, Berendsen HJC (1977). Numerical integration of cartesian equations of motion of a system with constraints: molecular dynamics of N-alkanes. J Comput Phys.

[115] Piana S (2012). Evaluating the effects of cutoffs and treatment of long-range electrostatics in protein folding simulations. PLoS One.

[116] Berendsen HJC, Postma JPM, van Gunsteren WF, Dinola A, Haak JR (1984). Molecular Dynamics with coupling to an external bath. J Chem Phys.

[117] Loncharich RJ, Brooks BR, Pastor RW (1992). Langevin dynamics of peptides: the frictional dependence of isomerization rates of N-acetylalanyl-N’-methylamide. Biopolymers.

[118] Frisch, M. J. *et al*. *Gaussian 16, Revision B.01.* (Gaussian Inc., Wallingford CT, 2016).

[119] Szabo, A. & Ostlund, N. S. *Modern quantum chemistry: introduction to advanced electronic structure theory*. (Dover Publications, 1996).

[120] Cornell WD, Cieplak P, Bayly CI, Kollman PA (1993). Application of RESP charges to calculate conformational energies, hydrogen bond energies, and free energies of solvation. J Am Chem Soc.

[121] Wang JM, Wolf RM, Caldwell JW, Kollman PA, Case DA (2004). Development and testing of a general Amber force field. J Comput Chem.

[122] Lee B, Richards FM (1971). The interpretation of protein structures: estimation of static accessibility. J Mol Biol.

[123] McDonald IK, Thornton JM (1994). Satisfying hydrogen-bonding potential in proteins. J Mol Biol.

[124] Frishman D, Argos P (1995). Knowledge-based protein secondary structure assignment. Proteins: Struct, Funct, Genet.

[125] Lovell, S. C., Word, J. M., Richardson, J. S. & Richardson, D. C. The penultimate rotamer library. *Proteins: Struct Funct Genet* **40**, 389–408, https://doi.org/10.1002/1097-0134(20000815)40:3<389::AID-PROT50>3.0.CO;2-2 (2000). 10861930

