# Supplementary material for: Structural and dynamic origins of ESR lineshapes in spin-labeled GB1 domain: the insights from spin dynamics simulations based on long MD trajectories
Source: Sci Rep. 2020 Jan 22;10:957. doi: 10.1038/s41598-019-56750-y (PMC6976580; doi:10.1038/s41598-019-56750-y)
Supplement: Supplementary file 1 — Supplementary information. [file 41598_2019_56750_MOESM1_ESM.pdf]

Supplementary Information

Structural and dynamic origins of ESR lineshapes in spin-labeled GB1 domain: the insights from spin dynamics simulations based on long MD trajectories.

Sergei A. Izmailov,[1] Sevastyan O. Rabdano,[1] Zikri Hasanbasri,[2] Ivan S. Podkorytov,[1] Sunil Saxena,[2] Nikolai R. Skrynnikov[1,3*]

[1] Laboratory of Biomolecular NMR, St. Petersburg State University, St. Petersburg 199034, Russia.

[2] Department of Chemistry, University of Pittsburgh, Pittsburgh, PA 15260, USA

[3] Department of Chemistry, Purdue University, West Lafayette, IN 47907, USA

* Corresponding author (nikolai@purdue.edu)

1. Calculation of the ESR spectra

The time-dependent Hamiltonian $\mathbf{H}(t)$, as defined in Eq. (1), is constructed based on a given MD trajectory. For this purpose, we have used the g-tensor and hyperfine coupling tensor as reported by Oganesyan: $\mathbf{g}^{PAS}=(2.0085, 2.0063, 2.0025)$ and $\mathbf{A}^{PAS}=(6.0, 6.0, 36.6)$ G.[1] These results are consistent with the data from MTSSL-labeled proteins[2-3] and are representative of "generic" labeling site (i.e. the site that has certain degree of solvent exposure, but is not fully exposed). The PAS axes are standard: *x* is along the NO bond, *y* is in the plane of proxyl ring (orthogonal to *x*) and *z* is perpendicular to the plane of the proxyl ring (see Fig. 1 for visualization of PAS). Using the MD coordinates, we take *x* to be along the NO bond, then generate *z* via cross-product between *x* and the vector connecting two carbon atoms that are directly bonded to nitrogen, and finally generate *y* via cross-product of *x* and *z*. On the other hand, the laboratory frame in the MD simulation is the frame where atomic coordinates are defined, i.e. it has *x* axis along the vector [1, 0, 0], *y* axis along [0, 1, 0] and *z* axis along [0, 0, 1]. Having determined the PAS frame and the laboratory frame, we can calculate for each individual MD snapshot the transition matrix $\mathbf{R}(t)$, which connects these two frames. Thus, based on the MD trajectory we construct the time-dependent Hamiltonian matrix $\mathbf{H}(t)$, cf. Eq. (1) in the main text.

As a next step, we use $\mathbf{H}(t)$ to build the propagators $\mathbf{U}(t,t+\delta)$ as follows:

$$\mathbf{U}(t,t+\delta)=e^{i\mathbf{H}(t)\delta} \qquad \text{(S1).}$$

It is assumed that the step $\delta=1$ ps is sufficiently small so that the Hamiltonian $\mathbf{H}(t)$ does not change appreciably during this time interval. Therefore, Eq. (S1) can be viewed as a legitimate representation of the evolution operator (note that it differs slightly from the conventional definition of the evolution operator, but we find the form in Eq. (S1) somewhat more convenient). With this definition, the propagation of the spin density matrix $\boldsymbol{\sigma}(t)$ over the time interval $\delta=1$ ps is given by:

$$\boldsymbol{\sigma}(t+\delta)=\mathbf{U}^{-1}(t,t+\delta)\boldsymbol{\sigma}(t)\mathbf{U}(t,t+\delta) \qquad \text{(S2).}$$

Next, we calculate the "packet" propagators:

$$\mathbf{U}_{pack}(mT,(m+1)T)=\mathbf{U}(mT,mT+\delta)\mathbf{U}(mT+\delta,mT+2\delta)\cdots\mathbf{U}((m+1)T-\delta,(m+1)T) \qquad \text{(S3).}$$

The time interval T has been set to 50 ps and $m=0, 1, 2, \ldots$. Hence, we prepare the set of $\mathbf{U}_{pack}$ matrices, where the first one describes the evolution of the system from 0 to 50 ps, the second – from 50

to 100 ps, the third – from 100 to 150 ps, etc. The choice of $T = 50$ ps is motivated by the Nyquist theorem for sampling of the ESR free induction decay.

As a next step, we use $\mathbf{U}_{pack}$ matrices to describe the evolution of the system over extended time intervals:

$$\mathbf{U}_{ext}\left(l\Delta, l\Delta + kT\right) = \mathbf{U}_{pack}\left(l\Delta, l\Delta + T\right)\mathbf{U}_{pack}\left(l\Delta + T, l\Delta + 2T\right)\cdots\mathbf{U}_{pack}\left(l\Delta + (k-1)T, l\Delta + kT\right) \quad \text{(S4)},$$

where $\Delta = 5$ ns, $k = 1, 2, 3, \ldots$ and $l = 0, 1, 2, \ldots$. Thus the first series of matrices ($k = 1$) describes the evolution of the system from 0 to 0.05 ns, from 5 to 5.05 ns, from 10 to 10.05 ns, etc. The second series of matrices ($k = 2$) describes the evolution from 0 to 0.1 ns, from 5 to 5.1 ns, from 10 to 10.1 ns, etc. One such series of propagators is schematically illustrated in Fig. S1 using red arrows.

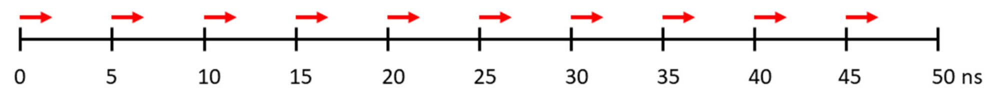

Figure S1. A series of propagators $\mathbf{U}_{ext}$ (schematically indicated by red arrows) with a fixed value of k. For the sake of illustration, this graph represents a short 50-ns MD trajectory. We comment on the meaning of $\Delta = 5$ ns below.

Finally, we average all propagators $\mathbf{U}_{ext}$ with a given value of k:

$$\mathbf{\Gamma}\left(kT\right) = \frac{\sum_{l=0}^{N-1}\mathbf{U}_{ext}\left(l\Delta, l\Delta + kT\right)}{N} \quad \text{(S5)},$$

$$N = \left(\frac{L}{\Delta}\right) - ceil\left(\frac{kT}{\Delta}\right) + 1 \quad \text{(S6)},$$

where L is the length of the MD trajectory (assumed commensurate with $\Delta = 5$ ns) and the function $ceil(x)$ rounds the argument x upward to the nearest integer. The expressions Eqs. (S5, S6) hold for $kT < \Delta$ (illustrated in Fig. S1) as well as $kT \geq \Delta$; they correspond to a variant of a sliding time-window scheme.

Using the average propagators $\mathbf{\Gamma}\left(kT\right)$ we calculate the time-evolution of the spin-density matrix:

$$\boldsymbol{\sigma}\left(kT\right) = \mathbf{\Gamma}^{-1}\left(kT\right)\boldsymbol{\sigma}\left(0\right)\mathbf{\Gamma}\left(kT\right) = \mathbf{\Gamma}^{-1}\left(kT\right)\mathbf{S}_x\mathbf{\Gamma}\left(kT\right) \quad (k = 0, 1, 2, \ldots) \quad \text{(S7)},$$

where $\mathbf{S}_x$ refers to the *x*-component of the electron spin operator. This result is used as a basis to calculate the spectrum $s(\omega)$ according to Eqs. (4, 5).

At this point it is appropriate to comment on the choice of Δ. This parameter is essentially the stride used to generate U_{ext} matrices. In principle, choosing smaller Δ leads to better statistical properties of the result, but increases the computational time. For the system at hand, we satisfied ourselves that $\Delta = 5$ ns is the optimal setting. Specifically, we re-calculated all spectra using $\Delta = 0.5$ ns (results not shown) and found them to be identical to those obtained with $\Delta = 5$ ns, whereas the time required to compute a single spectrum increased sevenfold, from 5.5 hrs to 40 hrs. We note that the proper choice of Δ maybe be dependent on the details of the system and the length of the trajectory. The setting of this parameter needs to be tested to ensure good convergence properties of the calculations.

One additional possibility exists to improve statistical properties of the simulated spectra $s(\omega)$. While each of our MD trajectories is sufficiently long, the simulated nitroxide group does not sample different spatial orientations in a perfectly isotropic manner (this is just a consequence of finite simulation length). To improve on this aspect, for each trajectory we have generated a series of replica trajectories that differ from each other by a constant rotation. Specifically, multiple copies of the original trajectory have been generated such that the initial orientations of the NO bond cover 225 nodes uniformly distributed on a unit sphere.[4] Each of the copies is used to independently compute $s(\omega)$ and the results are subsequently averaged. We have found that the grid containing 225 nodes is adequate for the purpose of these analyses – use of finer grids does not change the shape of the spectrum. In principle, it is possible to enhance orientational sampling by introducing an additional dimension to the grid, corresponding to rotation about the NO bond. In practice, however, this proved to be unnecessary.

We have also undertaken additional series of simulations to probe the effect of small variation in the magnitude of tensors $\mathbf{g}$ and $\mathbf{A}$. Specifically, we used the values $\mathbf{g}^{PAS}=(2.00841, 2.00604, 2.00223)$, $\mathbf{A}^{PAS}=(5.25, 5.25, 36.18)$ G, which have been reported for proxyl spin label in glycerol glass (believed to be a good model of protein exterior).[5] This substitution led to a small, but appreciable bias in the simulated spectra, compromising the agreement with the experimental data. Furthermore, we have also tested the values $\mathbf{g}^{PAS}=(2.00905, 2.00617, 2.00227)$, $\mathbf{A}^{PAS}=(4.85, 4.82, 33.26)$ G, which were reported for proxyl spin label in *ortho*-terphenyl (believed to be a good model of protein interior).[5] In this case, the simulated spectra were clearly off the mark. Therefore, we stayed with the initial choice of $\mathbf{g}^{PAS}$ and $\mathbf{A}^{PAS}$, which showed the best results in our calculations. Nevertheless, one should bear in mind that small

environment-dependent variations in $\mathbf{g}^{PAS}$ and $\mathbf{A}^{PAS}$ are in principle possible. This may be relevant for future efforts to achieve quantitative accuracy in interpreting ESR spectra of spin-labeled proteins.

Finally, we have also explored the effect from two additional interactions: quadrupolar interaction for ^{14}N nuclear spin $I=1$ and the Zeeman interaction for the same nuclear spin. The corresponding terms have been added to the time-dependent Hamiltonian $\mathbf{H}(t)$, Eq. (1). The parameters of the quadrupolar tensor were taken from the paper by Savitsky *et al.*[5] Using this extended Hamiltonian, we repeated the calculations of ESR spectra for all spin-labeled GB1 variants investigated in this work. It was found that including these two interactions has no appreciable effect on the appearance of the simulated spectra. This is expectable since both of them are small compared to the hyperfine coupling, which dominates spin dynamics of ^{14}N and, more broadly, spin dynamics of the coupled two-spin system.

2. Processing of the MD data

2.1. Determination of tumbling time

The following procedure has been used to extract τ_{rot} from the MD trajectory. First, we parameterized the rotation of GB1 molecule from frame i to frame $i+1$ along the entire trajectory. To this end, we superimposed the scaffold of GB1 from frame i onto the scaffold of GB1 from frame $i+1$ (specifically, we superimposed C$^{\alpha}$ atoms belonging to the secondary structure, where secondary structure was defined based on crystallographic coordinates of the protein). This operation can be represented as a combination of translation of the center of mass (of no interest for us) and rotation. In this manner we determined the matrices $\mathbf{\Omega}_{i,i+1}$, describing the step-wise reorientational motion of GB1. As a next step, we considered the set of 225 vectors of unit length $\mathbf{v}_0$ providing optimal sampling of a unit sphere.[4] For each of these vectors we apply consecutive rotations $\mathbf{\Omega}_{i,i+1}$ and thus construct vector trajectory $\mathbf{v}(t)$:

$$\mathbf{v}(t_k)=\mathbf{\Omega}_{k-1,k}\cdots\mathbf{\Omega}_{1,2}\mathbf{\Omega}_{0,1}\mathbf{v}_0 \qquad \text{(S8).}$$

In turn, this vector trajectory is used to construct the temporal correlation function:

$$g(\tau)=\langle P_2(\cos\theta)\rangle \qquad \text{(S9)}$$

where $P_2(x)$ is a second-order Legendre polynomial, θ is the angle between vector orientations $\mathbf{v}(t_m)$ and $\mathbf{v}(t_n)$, and angular brackets denote averaging over all pairs m,n such that $t_m-t_n=\tau$. The correlation functions $g(\tau)$ calculated for 225 different initial orientations $\mathbf{v}_0$ are then averaged with the prescribed weights,[4] resulting in the average correlation function $G(\tau)$. Finally, the obtained $G(\tau)$ is fitted with a single exponential, thus allowing one to extract the correlation time τ_{rot}. The quality of the fitting is very

good, indicating that this procedure is well suited to obtain an accurate measure of isotropic reorientational diffusion in a protein with moderate anisotropy, such as GB1.

As described in the main text, the extracted value of τ_{rot} is in good agreement with the experimental results, as well as theoretical predictions. It is important to realize, however, that this agreement involves a certain element of error compensation. On one hand, it is known that the standard TIP3P model employed in our simulation underestimates shear viscosity of water.[6] On the other hand, the commonly used NPT ensemble with collision frequency γ = 2 ps^{-1} effectively raises solvent viscosity.[7] The fortuitous compensation of these two factors leads to τ_{rot} value in line with expectations.

Note that, in principle, we do not need to rely on error cancelation to obtain correct τ_{rot}. Using one of more advanced water models in conjunction with NVE ensemble offers a good practical solution to this problem.[8] Furthermore, good results can be obtained using NPT ensemble with the thermostat developed by Bussi and co-workers.[9-10] Importantly, the length of MD simulations accessible through GPU computing greatly exceeds the typical time scale of protein tumbling: in our case, each trajectory is nearly 4 orders of magnitude longer than τ_{rot}. All of this taken together makes it possible to model protein tumbling with a quantitative accuracy.

2.2. Adjustment of tumbling time

The goal here is to build a pseudo-trajectory, which would differ from the original trajectory in only one characteristic – namely, the rate of the overall protein tumbling. For this purpose, we have developed the algorithm that is described below. In what follows, protein coordinates from the i-th frame in the original MD trajectory are denoted $\mathbf{v}_i$ and the corresponding coordinates in the pseudo-trajectory are denoted $\mathbf{V}_i$. The transformation that superimposes $\mathbf{v}_i$ onto $\mathbf{V}_j$ via C$^\alpha$ atoms in the secondary-structure regions is denoted $\mathbf{\Pi}\left(\mathbf{v}_i \rightarrow \mathbf{V}_j\right)$. We are mainly interested in the rotational portion of this transformation, which is denoted $\mathbf{R}\left(\mathbf{v}_i \rightarrow \mathbf{V}_j\right)$.

To construct the pseudo-trajectory, we assume that $\mathbf{V}_0 = \mathbf{v}_0$ and formulate the recursive algorithm to calculate $\mathbf{V}_{i+1}$ from $\mathbf{v}_{i+1}$ and $\mathbf{V}_i$.

1. We find the transformation $\mathbf{\Pi}\left(\mathbf{v}_i \rightarrow \mathbf{V}_i\right)$ that superimposes $\mathbf{v}_i$ onto $\mathbf{V}_i$.

2. We apply this transformation to $\mathbf{v}_{i+1}$:

$$\mathbf{V}_{ref} = \mathbf{\Pi}\left(\mathbf{v}_i \rightarrow \mathbf{V}_i\right)\mathbf{v}_{i+1} \qquad \text{(S10).}$$

3. We use the results to obtain the transformation $\mathbf{\Pi}\left(\mathbf{V}_i \rightarrow \mathbf{V}_{ref}\right)$, including the rotational matrix, $\mathbf{R}\left(\mathbf{V}_i \rightarrow \mathbf{V}_{ref}\right)$. This latter matrix can be parameterized via the direction of rotation axis, (θ,φ), and the amplitude of rotation, ω. We redefine this matrix by scaling the amplitude of rotation, $\omega_\lambda = \lambda\omega$. The resulting scaled rotation is denoted $\mathbf{R}_\lambda\left(\mathbf{V}_i \rightarrow \mathbf{V}_{ref}\right)$.

4. To generate the coordinates $\mathbf{V}_{i+1}$, we begin with the original snapshot $\mathbf{v}_{i+1}$, superimpose it onto $\mathbf{V}_i$ and then apply the scaled rotation:

$$\mathbf{V}_{i+1} = R_\lambda\left(\mathbf{V}_i \rightarrow \mathbf{V}_{ref}\right)\mathbf{\Pi}\left(\mathbf{v}_{i+1} \rightarrow \mathbf{V}_i\right)\mathbf{v}_{i+1} \quad \text{(S11).}$$

Using the scaling coefficient $\lambda < 1.0$ we reduce the amplitude of protein rotation at each 1-ps step in the trajectory. Consequently, the pseudo-trajectory is characterized by slower overall tumbling of the protein molecule. Conversely, using $\lambda > 1.0$ leads to faster than original tumbling. Bear in mind that applying the scaling factor λ to the amplitudes of elementary rotations results in scaling of τ_{rot} by the factor λ^2.[11] Clearly, the choice of λ is subject to certain limitations. Large λ is problematic since one can no longer assume that $\mathbf{H}(t)$ remains constant during the time interval $\delta = 1$ ps. In the case of very small λ, one has to pay special attention to the orientational sampling scheme, see SI section 1. In particular, high-quality sampling is necessary for the mutants with restricted R1 dynamics, such as F30R1 (see Fig. S12).

3. Redfield-theory calculations

Redfield formalism that we have used in this paper to calculate ESR spectra of spin-labeled GB1 is fundamentally the standard Redfield formalism. However, there are certain aspects of these calculations that deserve a special comment. A brief summary of our computational procedure is presented below.

We treat here the system that is comprised of two spins: the electron spin S=1/2 and ^{14}N nuclear spin I=1. We begin by dividing the Hamiltonian, Eq. (1), into two parts: time-independent part $\mathbf{H}_0$, which consists of the isotropic components of all relevant interactions, and time-dependent part $\mathbf{H}_1(t)$, which consists of the anisotropic terms and is sensitive to reorientation of the proxyl ring. The latter can be represented in a standard form:

$$\mathbf{H}_1(t) = \sum_\alpha \sum_{m=-2}^{2} \mathbf{A}_m^\alpha F_m^\alpha(t) \quad \text{(S12),}$$

where $\mathbf{A}_m^\alpha$ are spin operators and $F_m^\alpha(t)$ are spatial functions. Index α indicates the type of interaction and m enumerates the components of the respective (second-rank) tensor.

Initially, we make use of the conventional operator basis $\mathbf{B}_i = |m_S, m_I\rangle\langle m'_S, m'_I|$ to fill out the matrix of the Liouvillian $\mathbf{L}_0$ (36x36):

$$\mathbf{L}_0(i,j) = Tr\left\{\mathbf{B}_i^\dagger\left[\mathbf{H}_0, \mathbf{B}_j\right]\right\} \qquad \text{(S13).}$$

Next, we diagonalize this matrix, thus arriving at the eigenoperators $\mathbf{V}_i$ and the corresponding eigenvalues ω_i (transition frequencies):

$$\mathbf{L}_0\mathbf{V}_i = \omega_i\mathbf{V}_i \qquad \text{(S14).}$$

Since $\mathbf{L}_0$ is Hermitian, its eigenoperators $\mathbf{V}_i$ can be rendered orthogonal (including the case of degenerate eigenvalues). Note that state-of-the-art routines for diagonalizing Hermitian matrices automatically produce $\mathbf{V}_i$ that are orthonormal. Consequently, the basis $\mathbf{V}_i$ can be used to conveniently represent $\mathbf{A}_m^\alpha$, as well as spin-density matrix of the system, $\boldsymbol{\sigma}(t)$:

$$\mathbf{A}_m^\alpha = \sum_p Tr\left\{\mathbf{V}_p^\dagger\mathbf{A}_m^\alpha\right\}\mathbf{V}_p \qquad \text{(S15),}$$

$$\boldsymbol{\sigma}(t) = \sum_q s_q(t)\mathbf{V}_q \qquad \text{(S16).}$$

With these provisions, Redfield equation can be formulated as follows:

$$\frac{d}{dt}\left(s_k(t) - s_k^{eq}\right) = -i\omega_k\left(s_k(t) - s_k^{eq}\right) - \sum_l \Re_{kl}\left(s_l(t) - s_l^{eq}\right) \qquad \text{(S17),}$$

$$\Re_{kl} = \sum_{\alpha,\alpha'}\sum_{m,m'}\sum_{p,q} Tr\left\{\mathbf{V}_p^\dagger A_m^\alpha\right\} Tr\left\{\mathbf{V}_q^\dagger A_{m'}^{\alpha'\dagger}\right\} Tr\left\{\mathbf{V}_k^\dagger\left[\mathbf{V}_p,\left[\mathbf{V}_q,\mathbf{V}_l\right]\right]\right\} j_{m,m'}^{\alpha,\alpha'}\left(\omega_q\right) \qquad \text{(S18),}$$

$$j_{m,m'}^{\alpha,\alpha'}(\omega) = \int_0^\infty g_{m,m}^{\alpha,\alpha'}(\tau)\exp(-i\omega\tau)\,d\tau \qquad \text{(S19),}$$

$$g_{m,m'}^{\alpha,\alpha'}(\tau) = \left\langle F_m^\alpha(t)\,F_{m'}^{\alpha'*}(t+\tau)\right\rangle \qquad \text{(S20).}$$

Here $g_{m,m'}^{\alpha,\alpha'}(\tau)$ are temporal correlation functions that can be computed based on the MD data by means of fast Fourier transformation.[12] Note that $j_{m,m'}^{\alpha,\alpha'}(\omega)$ contain both real and imaginary part (corresponding to spin relaxation and dynamic frequency shift, respectively[13]). Note also that Eq. (S17) can be solved in a straightforward manner, yielding the complete set of time-dependent coefficients $s_k(t)$. In turn, these results can be used to generate the ESR free induction decay:

$$FID(t) = \sum_k s_k(t)\,Tr\left\{\mathbf{V}_k\mathbf{S}_+\right\} \qquad \text{(S21).}$$

Finally, $FID(t)$ can be converted into the calculated ESR spectrum as discussed in the main text.

Two aspects of the above algorithm deserve a special comment. First, the Hamiltonian $\mathbf{H}_0$ is not limited to the Zeeman term, but must also include the isotropic component of the hyperfine interaction. Second, special caution is needed when making use of the Redfield-theory secular approximation. For example, one has to bear in mind that ^{14}N Larmor frequency in the X-band measurements is much smaller than the hyperfine interaction. We have chosen not to invoke the secular approximation at all and instead solve Eq. (S17) with full Redfield matrix: 36x36 in the case of R1^{14N} or 16x16 in the case of R1^{15N}. The computational cost associated with this approach is minimal.

4. Supplemental figures

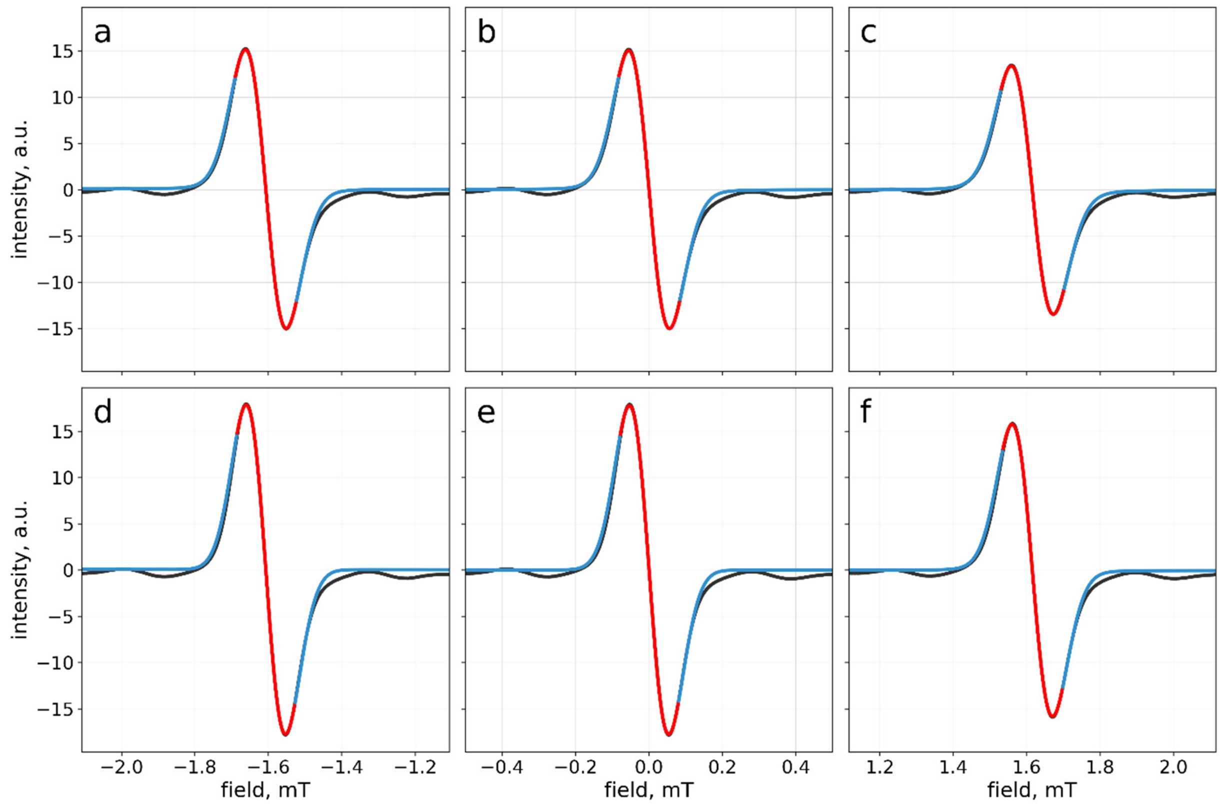

Figure S2. Simultaneous fitting of the six spectral lines as found in the experimental spectra of air-equilibrated sample of free MTSSL (a – low-field line, b – central line, c – high-field line) and degassed sample of free MTSSL (d – low-field line, e – central line, f – high-field line). Color scheme: black curve – experimental lineshape, red curve – portion of the fitted lineshape within the fitting range, blue curve – portion of the fitted lineshape outside the fitting range. Each peak is fitted with its individual Voigt contour assuming the following combination of the Lorentzian and Gaussian contributions: (a) ($L_{(1)}$+L_{O2},G); (b) ($L_{(0)}$+L_{O2},G); (c) ($L_{(-1)}$+L_{O2},G); (d) ($L_{(1)}$,G); (e) ($L_{(0)}$,G); (f) ($L_{(-1)}$,G). The entire collective fitting procedure, therefore, employs five essential fitting parameters corresponding to $L_{(1)}$, $L_{(0)}$, $L_{(-1)}$, L_{O2}, and G. Further details are described in Materials & Methods. For convenience, the spectra in the plot are referenced to H_0=0 .

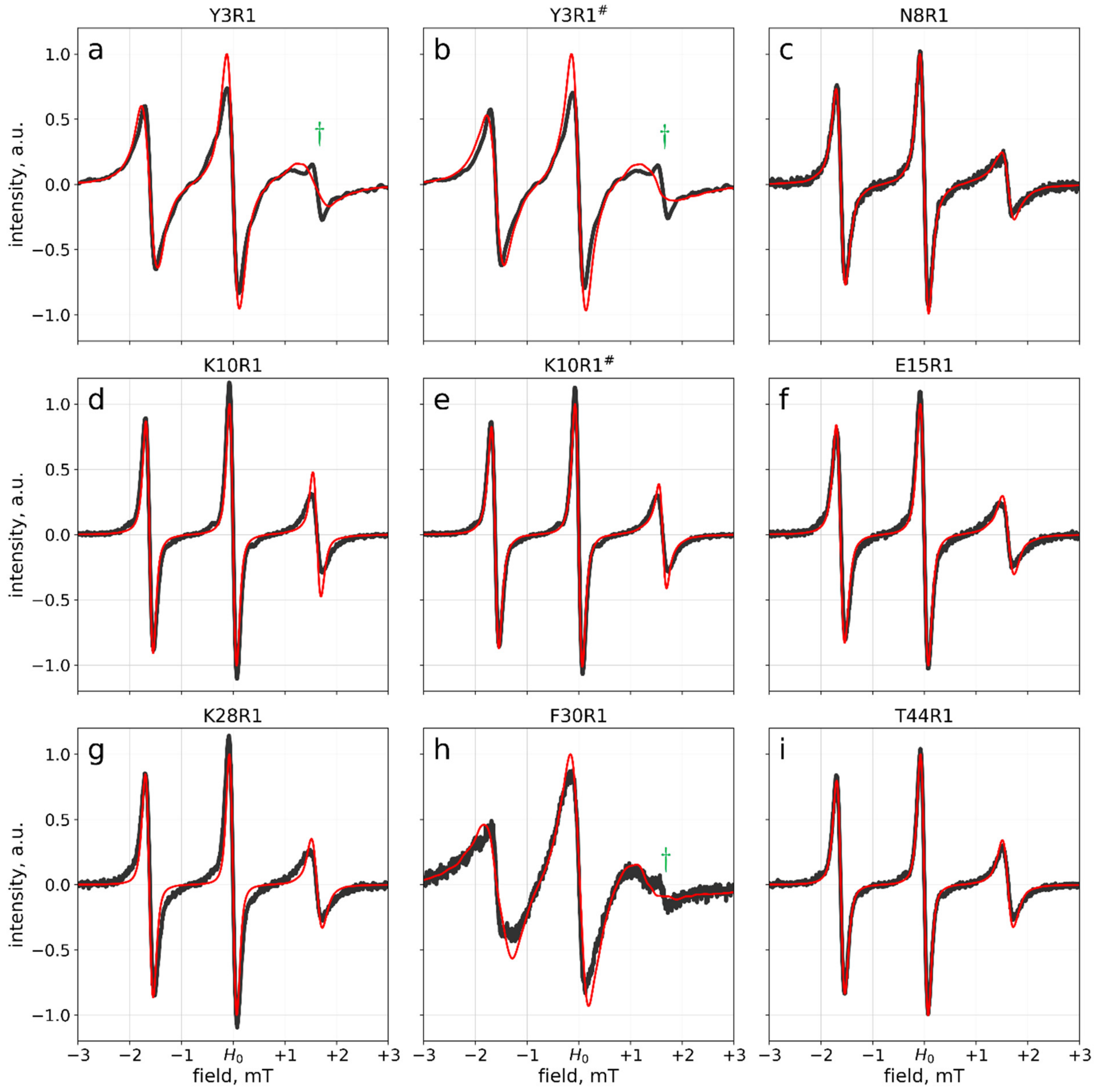

Figure S3. ESR spectra of seven spin-labeled mutants of GB1. The experimental and simulated spectra are plotted with broad black lines and thin red lines, respectively. The apparent difference in noise level, cf. panels (h) and (i), is a plotting artefact. Specifically, the data are scaled such that the maximum amplitude of the calculated signal is set to 1.0 (providing good view of all spectra). In the case of broad low-intensity spectra, such as F30R1, this representation emphasizes the presence of noise. The superscript # is used to indicate duplicate MD trajectories of Y3R1 and K10R1. The overall agreement between the calculations and experiment is good across the board. However, in the case of Y3R1 and F30R1, the simulation may not be fully representative of the actual state of the sample (cf. sharp spectral features marked in the plot by green daggers; see discussion in the main text). In the case of K28R1, the observed deviations between the experiment and the simulations may stem from the multicomponent character of the experimental spectrum.

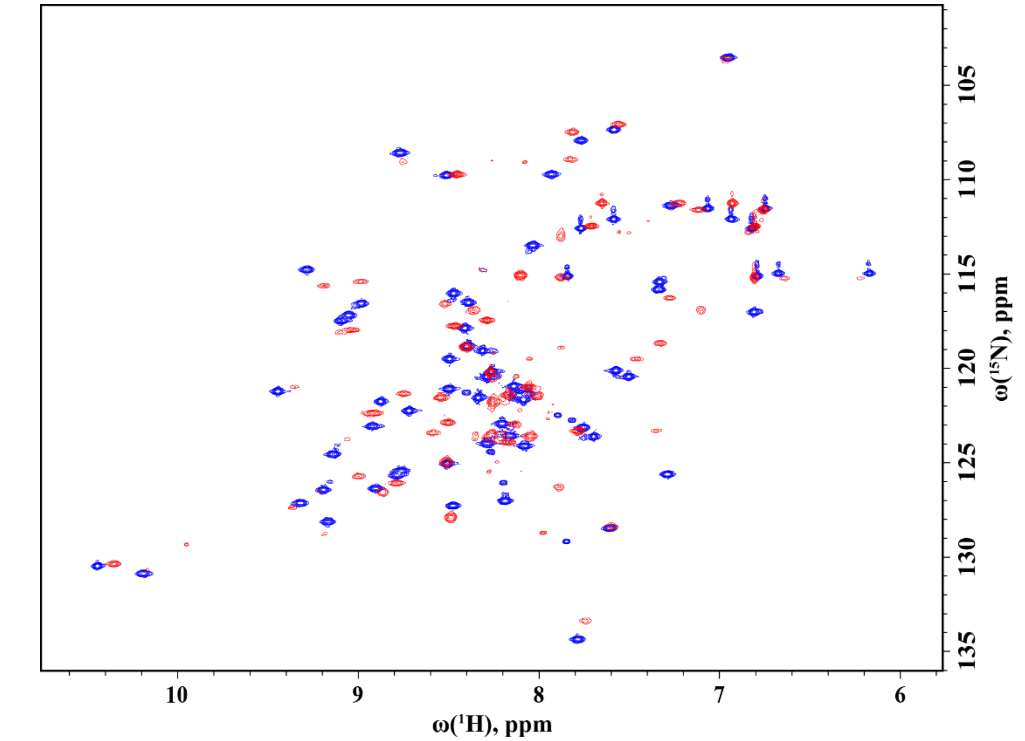

Figure S4. Superposition of ^{1}H,^{15}N HSQC maps from ^{15}N-enriched samples of GB1 F30C (blue) and F30R1 reduced by overnight incubation with 10 mM ascorbic acid (red). Contour levels are chosen such as to allow direct comparison between the two spectra. The spectrum of F30R1 re-acquired after 20 days was unchanged compared to the one shown in this plot.

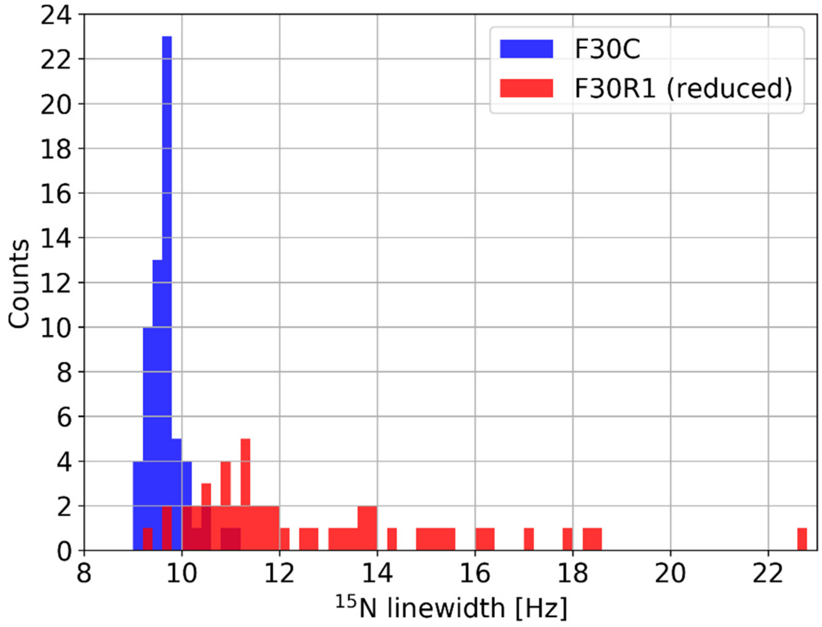

Figure S5. Histogram of ^{15}N linewidths in the HSQC spectral maps from GB1 F30C (blue) and F30R1 reduced by application of ascorbate (red). The data were obtained by reprocessing the data in Fig. S4 (without the window function in the ^{15}N dimension) and fitting the resulting peak shapes using the routine nlinLS from the package NMRPipe.[14] The comparison includes both backbone and side-chain resonances, but is limited to well-resolved peaks with substantial intensity.

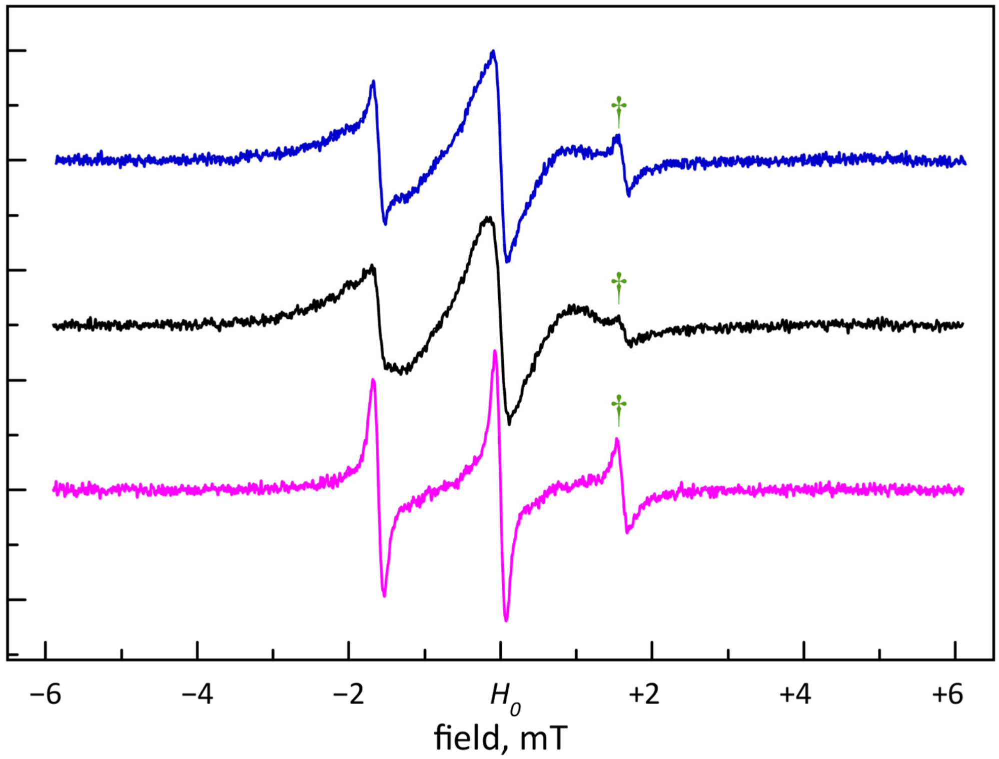

Figure S6. CW ESR spectra of three different GB1 F30R1 preparations: standard MTSSL labeling protocol, see Materials & Methods (upper trace, blue curve); standard MTSSL labeling protocol, followed by additional round of ion exchange chromatography (middle trace, black curve; also shown in Fig. S3h); MTSSL labeling protocol in solution with 8M urea (lower trace, magenta curve). The sharp components, nicely visible at high field, are marked by green daggers (note that these features are too broad to be attributable to free $MTSSL_{\Delta}$, which could conceivably appear in the sample due to reduction of the disulfide bond).

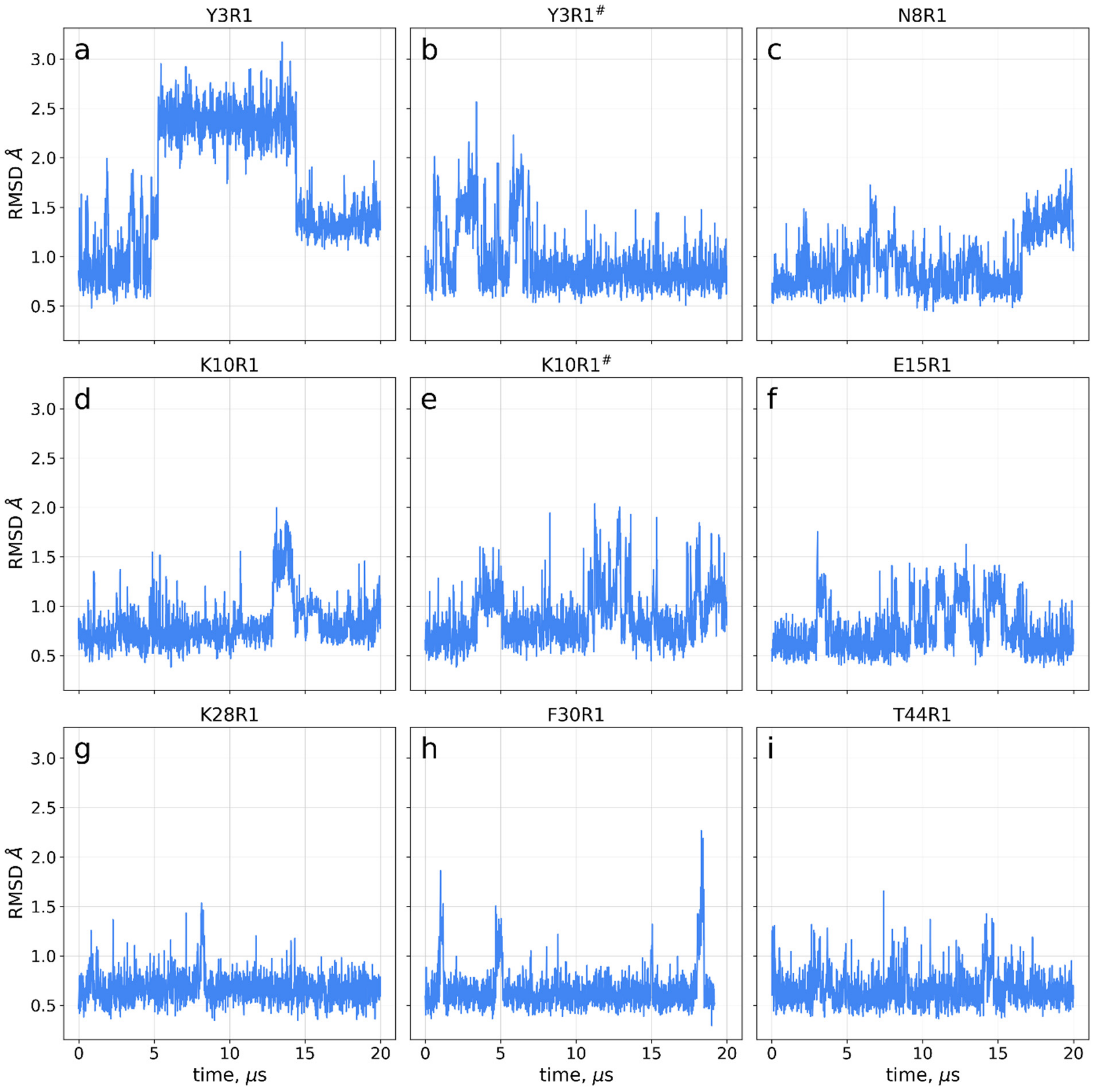

Figure S7. Root-mean-square deviation (*rmsd*) traces for all MD simulations of spin-labeled GB1. For each of the trajectories, *rmsd* is computed relative to the respective starting crystallographic structure (see Materials & Methods) using a fixed subset of backbone C^{α} atoms corresponding to the secondary-structure regions in the said crystallographic structure.

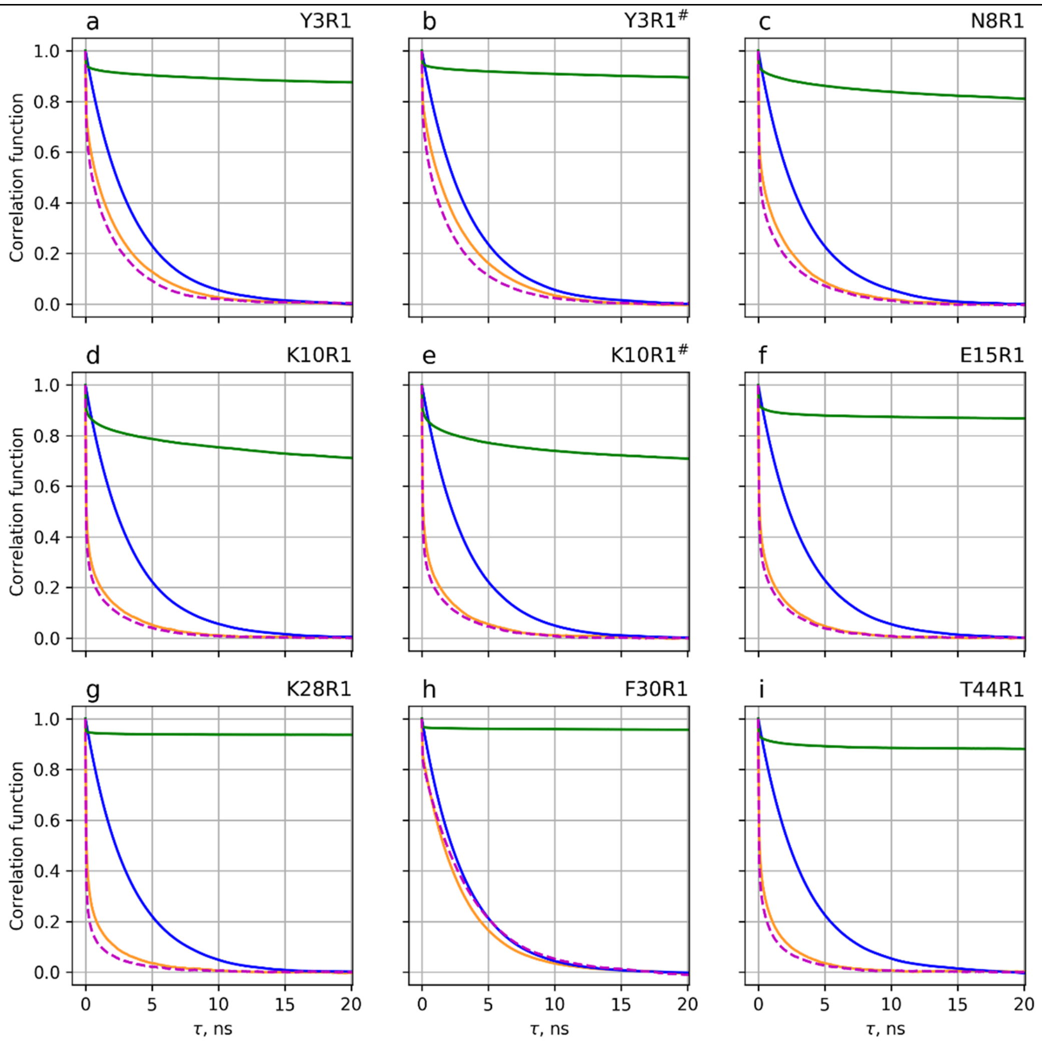

Figure S8. Temporal auto-correlation functions for selected vectors in the MD trajectories of spin-labeled GB1 (see SI section 2.1 for definitions). The choice of vectors and the algorithm to generate the correlation functions are as follows. (*Orange curve*) Correlation function $g(\tau)$ representing the reorientation of the NO bond from the R1 proxyl ring. (*Dashed magenta curve*) Correlation function $g(\tau)$ representing the reorientation of the normal to the R1 proxyl ring. Given the tensors $\mathbf{A}^{PAS}$ and $\mathbf{g}^{PAS}$, see SI section 1, this vector is the best predictor of dynamic averaging in the ESR spectrum. Note that it typically experiences more motion than the NO vector because of its greater sensitivity to χ_5 dynamics. (*Blue curve*) Correlation function $G(\tau)$ representing the overall tumbling of the protein; the calculation uses 100 vectors that are optimally distributed on the surface of the unit sphere.[4] (*Green curve*) Correlation function $g(\tau)$ representing the reorientation of $C^{\alpha}C^{\beta}$ bond from R1 residue as seen in the molecular frame of reference. To implement the transition from laboratory to molecular frame, protein coordinates from all MD frames are superimposed via C^{α} atoms belonging to the secondary-structure regions.

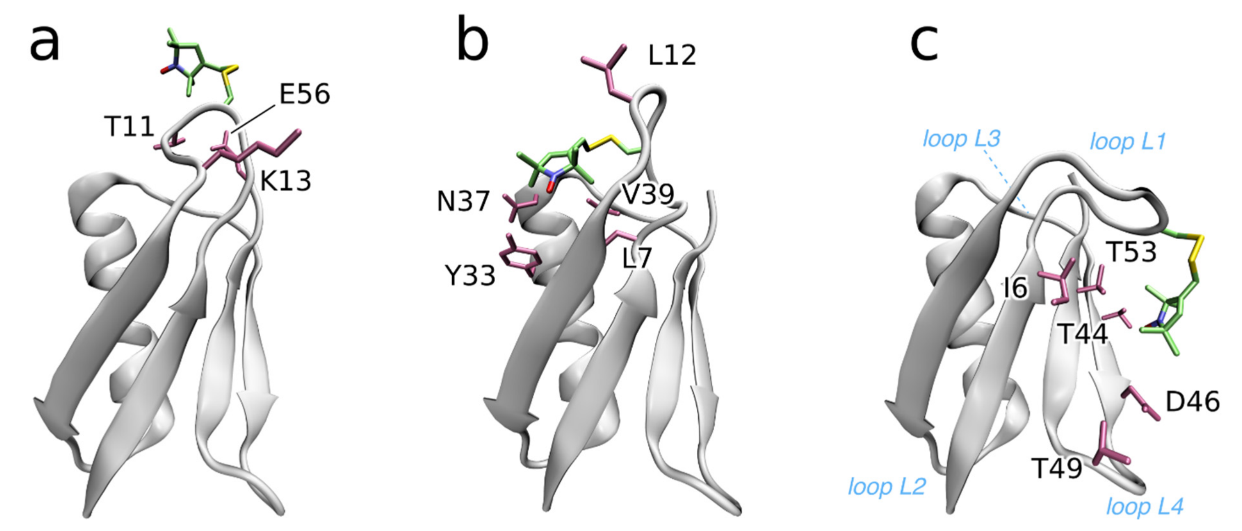

Figure S9. Three selected snapshots from the trajectory GB1 K10R1[#] representative of states A, B, and C (see main text for discussion). The snapshots correspond to moments in time 0.0, 7.0, and 20.0 μs.

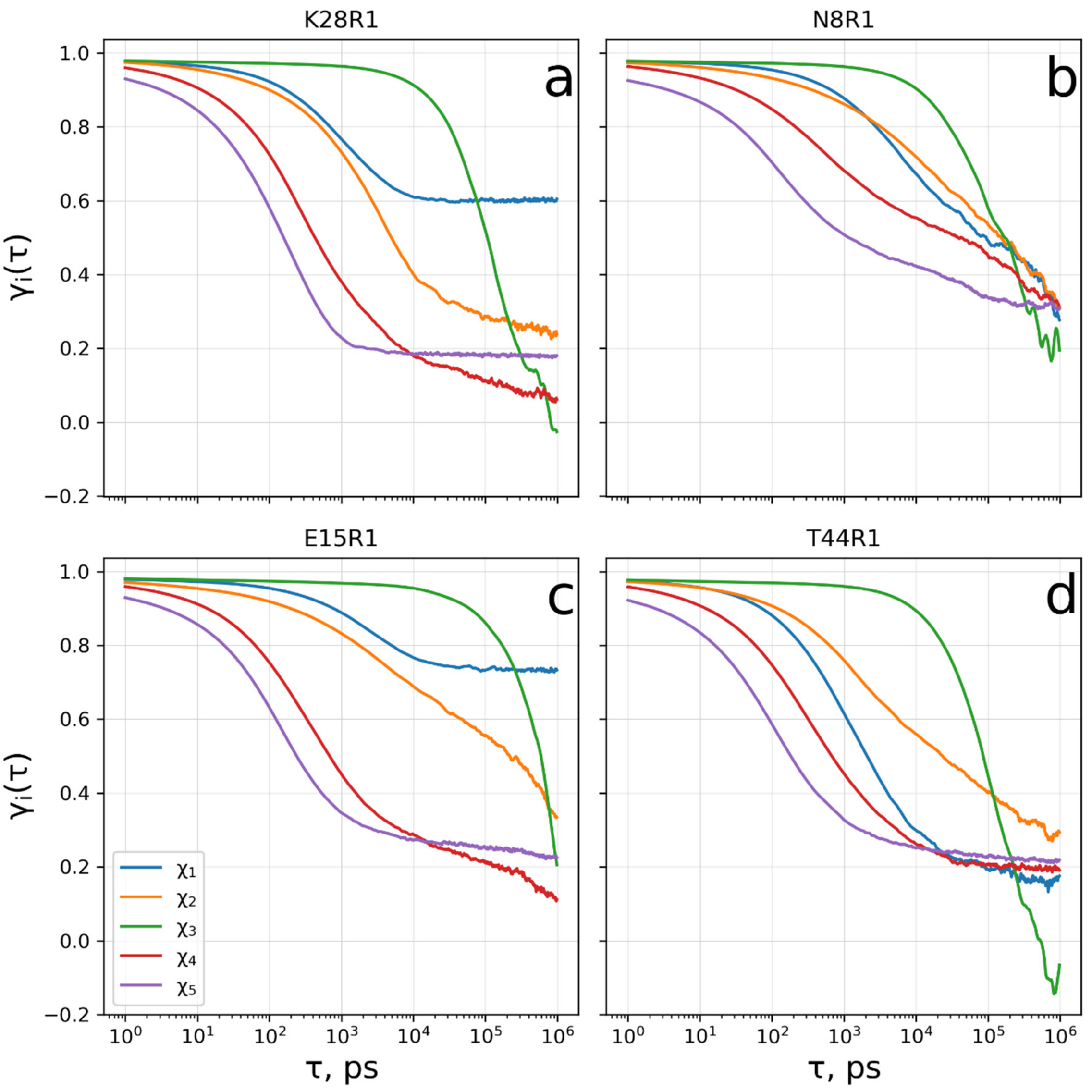

Figure S10. Temporal correlation functions $\gamma_i(\tau)$ characterizing torsional-angle dynamics of R1 tag in (a) K28R1, (b) N8R1, (c) E15R1 and (d) T44R1 trajectories. The functions are computed using the formula $\gamma_i(\tau) = \langle \cos \Delta\chi_i(\tau) \rangle$, where $\Delta\chi_i(\tau) = \chi_i(t+\tau) - \chi_i(t)$ and the angular brackets denote time-average over the entire length of the trajectory. The non-zero plateau values of $\gamma_i(\tau)$ curves reflect the conformational preferences of the R1 tag in the given trajectory. The failure to reach a plateau reflects lack of convergence. This is particularly relevant for γ_3 (green curve) since χ_3 jumps occur relatively infrequently during the course of the simulation, see text.

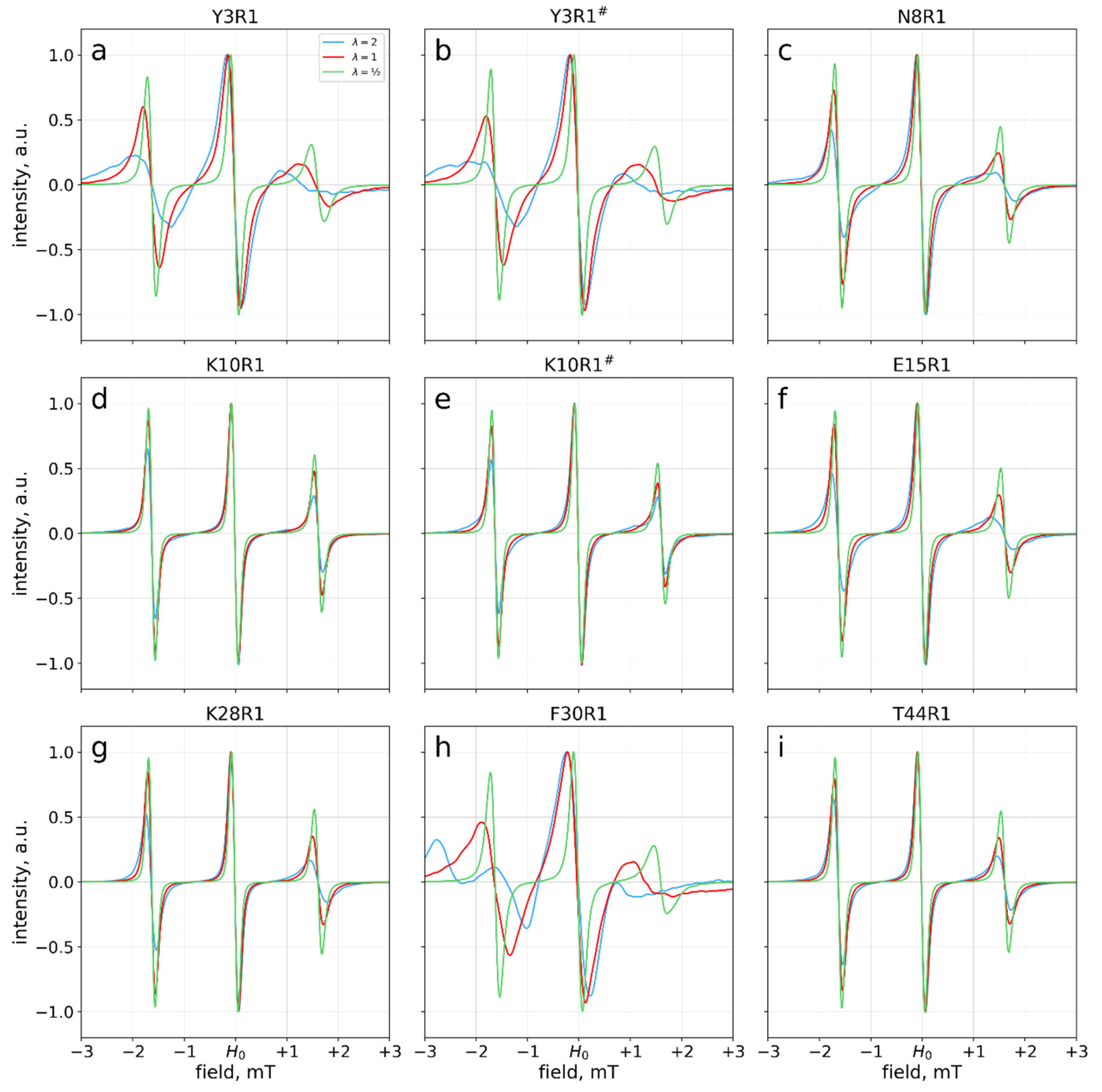

Figure S11. The simulated ESR spectra from nine MD trajectories of spin-labeled variants of GB1: (*red curves*) standard calculations identical to those shown in Fig. S3; (*green curves*) the trajectories have been pre-processed to increase the rate of the protein overall tumbling, $\lambda = 2.0$; (*blue curves*) the trajectories have been pre-processed to lower the rate of the protein overall tumbling, $\lambda = 0.5$. The details of the MD processing scheme are described in the main text and SI section 2.2. In this graph, each spectrum is normalized according to the height of its central line.

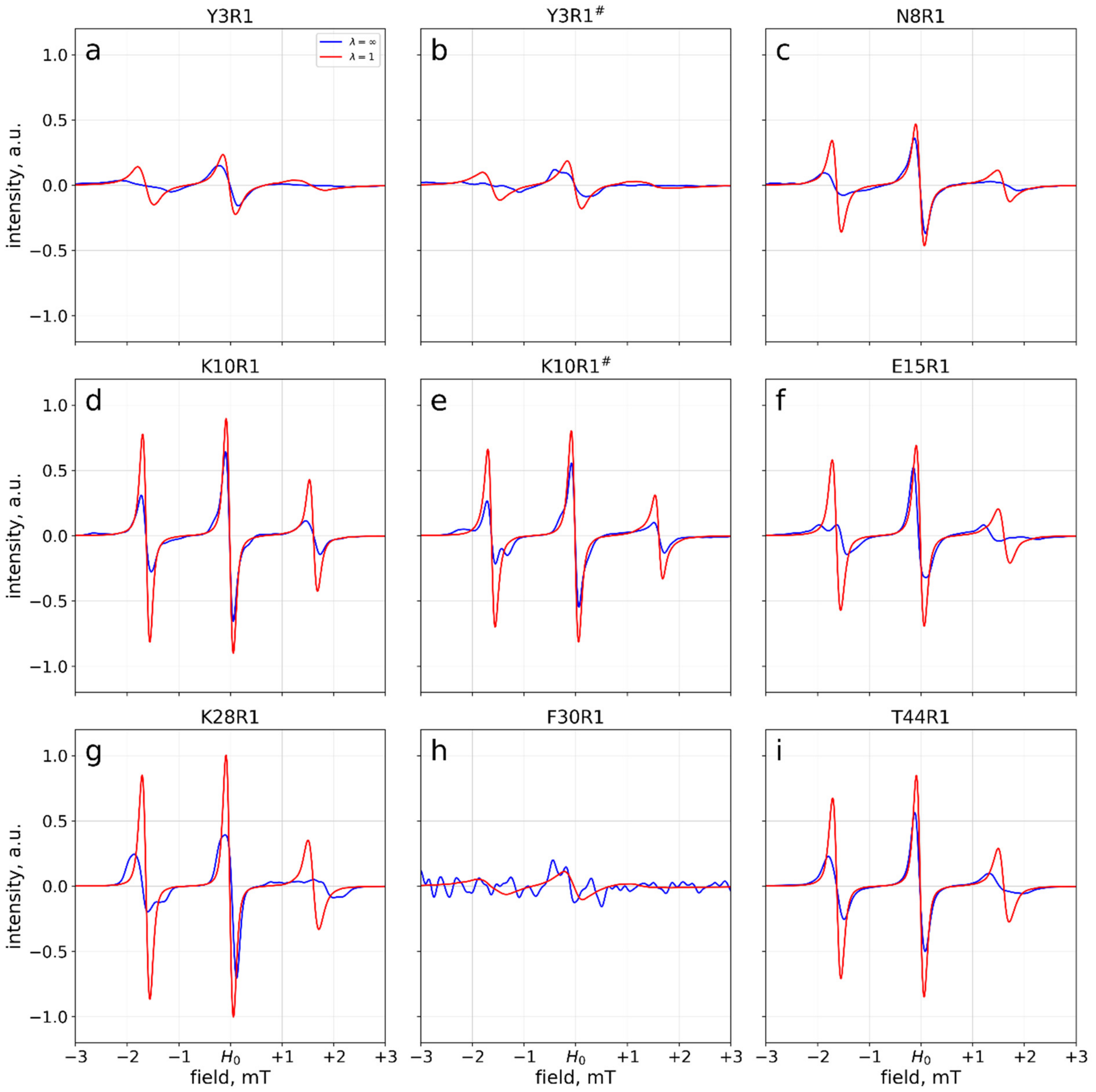

Figure S12. The simulated ESR spectra from nine MD trajectories of spin-labeled variants of GB1: (*red curves*) standard calculations identical to those shown in Fig. S3; (*blue curves*) the trajectories have been pre-processed to quench the overall tumbling, $\lambda = \infty$. For the mutants with restricted R1 dynamics, such as F30R1, the $\lambda = \infty$ spectrum approaches the limiting case of a static powder spectrum. In this situation, it is important to employ a high-quality sampling scheme encompassing the three orientational degrees of freedom of the spin-labeled protein. The scheme used in our work is relatively sparse (see SI section 1 for discussion) and hence gives rise to appreciable computational artefacts, such as seen in panel (h). Further consideration of $\lambda = \infty$ spectra, which are relevant for large proteins, microcrystalline proteins, precipitated proteins, membrane proteins in micelles and lipid bilayers, etc., is deferred to future work. Unlike in Figs. S3 and S11, the individual spectra in this graph are not normalized (and hence can be compared to each other in terms of signal intensities).

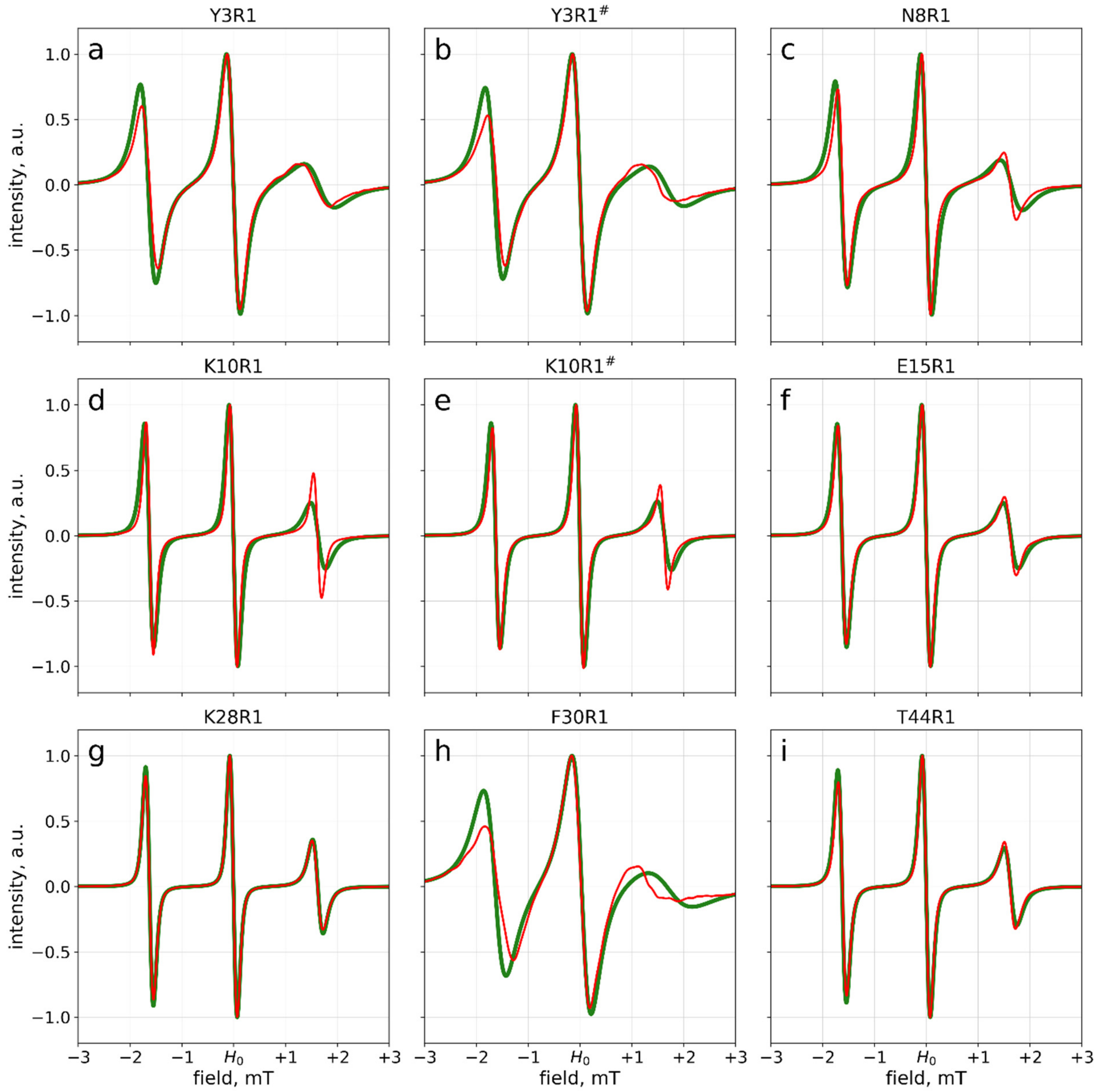

Figure S13. ESR spectra of seven spin-labeled mutants of GB1 calculated via MD-based direct propagation scheme (red lines, same as in Fig. S3) or, alternatively, Redfield formalism (green lines). The small-to-moderate deviations between the two methods can be attributed to the following factors. First, for samples such Y3R1 and F30R1, the Redfield theory is pushed to the limits of its applicability (see the main text for discussion). Second, some of the trajectories feature rare transitions between distinctive conformational states (e.g. K10R1 discussed in the main text). This means that MD statistics is essentially inadequate. The two methods, the direct propagation scheme and Redfield scheme, have different properties with respect to statistical sampling of the MD data. As a consequence, they respond differently to the insufficient statistics. This is likely a contributing factor to the discrepancies between the two spectra in panel (d) as well as panel (e).

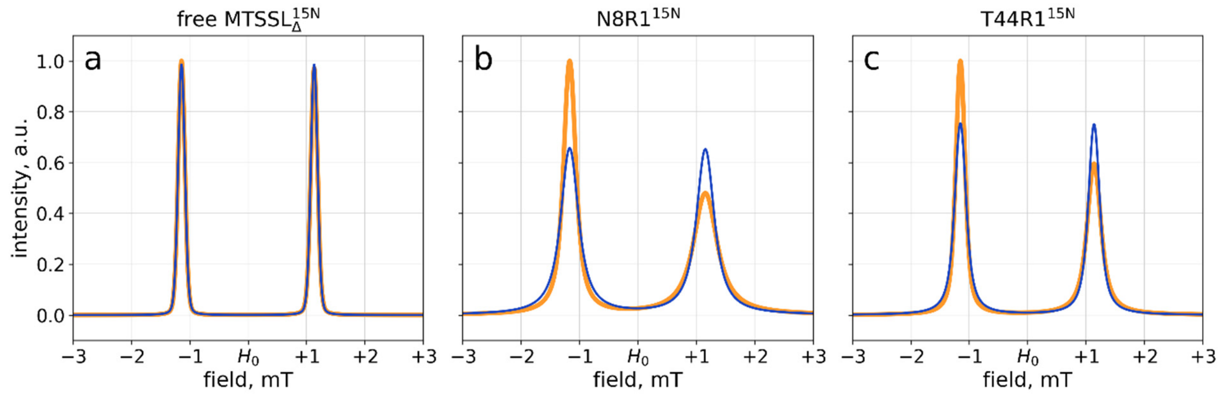

Figure S14. ESR spectra of free $MTSSL^{15N}_{\Delta}$, N8R1^{15N} and T44R1^{15N} calculated by means of the full-fledged Redfield-theory treatment (orange lines, same as in Fig. 4d-f), as well as the altered version of the same treatment, where the contributions to the Redfield matrix due to cross-correlations between the anisotropy of g-tensor and the hyperfine interaction have been set to zero (blue lines).

5. Supplemental references

1. Oganesyan, V. S. A general approach for prediction of motional EPR spectra from Molecular Dynamics (MD) simulations: application to spin labelled protein. *Phys. Chem. Chem. Phys.* **2011,** *13*, 4724-4737.
2. Gast, P.; Herbonnet, R. T. L.; Klare, J.; Nalepa, A.; Rickert, C.; Stellinga, D.; Urban, L.; Mobius, K.; Savitsky, A.; Steinhoff, H. J.; Groenen, E. J. J. Hydrogen bonding of nitroxide spin labels in membrane proteins. *Phys. Chem. Chem. Phys.* **2014,** *16*, 15910-15916.
3. Steinhoff, H. J.; Savitsky, A.; Wegener, C.; Pfeiffer, M.; Plato, M.; Mobius, K. High-field EPR studies of the structure and conformational changes of site-directed spin labeled bacteriorhodopsin. *Biochim. Biophys. Acta Bioenergetics* **2000,** *1457*, 253-262.
4. Fliege, J.; Maier, U. The distribution of points on the sphere and corresponding cubature formulae. *IMA J. Numer. Anal.* **1999,** *19*, 317-334.
5. Savitsky, A.; Dubinskii, A. A.; Plato, M.; Grishin, Y. A.; Zimmermann, H.; Mobius, K. High-field EPR and ESEEM investigation of the nitrogen quadrupole interaction of nitroxide spin labels in disordered solids: toward differentiation between polarity and proticity matrix effects on protein function. *J. Phys. Chem. B* **2008,** *112*, 9079-9090.
6. Angel Gonzalez, M.; Abascal, J. L. F. The shear viscosity of rigid water models. *J. Chem. Phys.* **2010,** *132*.
7. Loncharich, R. J.; Brooks, B. R.; Pastor, R. W. Langevin dynamics of peptides: the frictional dependence of isomerization rates of N-acetylalanyl-N'-methylamide. *Biopolymers* **1992,** *32*, 523-535.
8. Wong, V.; Case, D. A. Evaluating rotational diffusion from protein MD simulations. *J. Phys. Chem. B* **2008,** *112*, 6013-6024.
9. Bussi, G.; Donadio, D.; Parrinello, M. Canonical sampling through velocity rescaling. *J. Chem. Phys.* **2007,** *126*.
10. Hoffmann, F.; Mulder, F. A. A.; Schafer, L. V. Accurate methyl group dynamics in protein simulations with AMBER force fields. *J. Phys. Chem. B* **2018,** *122*, 5038-5048.
11. Haile, J. M. *Molecular Dynamics Simulation*. John Wiley & Sons, Inc.: New York, 1992.
12. Press, W. H.; Teukolsky, S. A.; Vetterling, W. T.; Flannery, B. P. *Numerical Recipes in C*. Cambridge University Press: Cambridge, 1992.
13. Werbelow, L.; London, R. E. Dynamic frequency shift. *Concepts Magn. Reson.* **1996,** *8*, 325-338.
14. Delaglio, F.; Grzesiek, S.; Vuister, G. W.; Zhu, G.; Pfeifer, J.; Bax, A. NMRPipe: a multidimensional spectral processing system based on unix pipes. *J. Biomol. NMR* **1995,** *6*, 277-293.
